# Integration of RNA-seq and ATAC-seq analyzes the effect of low dose neutron-γ radiation on gene expression of lymphocytes from oilfield logging workers

**DOI:** 10.3389/fchem.2023.1269911

**Published:** 2023-11-30

**Authors:** Weiguo Li, Gang Gao, Yan Pan, Ziqiang Wang, Jianlei Ruan, Li Fan, Yingjie Shen, Haiqing Wang, Mian Li, Pinhua Zhang, Lianying Fang, Jinghong Fu, Jianxiang Liu

**Affiliations:** ^1^ China CDC Key Laboratory of Radiological Protection and Nuclear Emergency, Chinese Center for Disease Control and Prevention, National Institute for Radiological Protection, Beijing, China; ^2^ School of Biomedical Sciences, Shandong First Medical University, Jinan, Shandong, China; ^3^ Safety and Environmental Protection Department, Shengli Logging Company, Sinopec Jingwei Co., LTD., Dongying, Shandong, China; ^4^ Dongying Center for Disease Control and Prevention, Dongying, Shandong, China; ^5^ School of Preventive Medicine, Shandong First Medical University Institute of Radiation Medicine, Shandong Academy of Medical Sciences, Jinan, Shandong, China

**Keywords:** oilfield logging workers, RNA-seq, ATAC-seq, neutron, differentially expressed gene, ribosome, pseudogene

## Abstract

**Objective:** Although radiation workers are exposed to much lower doses of neutron-γ rays than those suffered in nuclear explosions and accidents, it does not mean that their health is not affected by radiation. Lower doses of radiation do not always cause morphological aberrations in chromosomes, so more sophisticated tests must be sought to specific alterations in the exposed cells. Our goal was to characterize the specific gene expression in lymphocytes from logging workers who were continuously exposed to low doses of neutron-γ radiation. We hypothesized that the combination of cell type-specific transcriptomes and open chromatin profiles would identify lymphocyte-specific gene alterations induced by long-term radiation with low-dose neutron-γ-rays and discover new regulatory pathways and transcriptional regulatory elements.

**Methods:** Lymphocytes were extracted from workers who have been occupationally exposed to neutron-γ and workers unexposed to radiation in the same company. mRNA-seq and ATAC-seq (Assay for Transposase-Accessible Chromatin with high-throughput sequencing) were performed, followed integrative analysis to identify specific gene regulatory regions induced by neutron-γ radiation. A qPCR assay was then performed to verify the downregulation of RNA coding for ribosomal proteins and flow cytometry was used to detect ribosomal protein expression and cell cycle alterations.

**Results:** We identified transcripts that were specifically induced by neutron-γ radiation and discovered differential open chromatin regions that correlated with these gene activation patterns. Notably, we observed a downward trend in the expression of both differentially expressed genes and open chromatin peaks. Our most significant finding was that the differential peak upregulated in ATAC-seq, while the differential gene was downregulated in the ribosome pathway. We confirmed that neutron-γ radiation leads to transcriptional inhibition by analyzing the most enriched promoters, examining RPS18 and RPS27A expression by qPCR, and analyzing protein-protein interactions of the differential genes. Ribosomal protein expression and cell cycle were also affected by neutron-γ as detected by flow cytometry.

**Conclusion:** We have comprehensively analyzed the genetic landscape of human lymphocytes based on chromatin accessibility and transcript levels, enabling the identification of novel neutron-γ induced signature genes not previously known. By comparing fine-mapping of open chromatin and RNA reads, we have determined that neutron-γ specifically leads to downregulation of genes in the ribosome pathway, with pseudogenes potentially playing a crucial role.

## 1 Introduction

As neutron technology is increasingly being utilized in civilian industrial industries, such as nuclear power plants, prospecting, oil exploration, and aviation, there is a growing number of workers worldwide being exposed to neutrons. Although the neutron dose rates are relatively low, the stronger radiation-inducing ability of neutrons prompted researchers to investigate the health effects of low-dose neutron exposures on humans or animals. In 2019, NASA formed an interdepartmental working group dedicated to neutron dosimetry and neutron radiobiology, further emphasizing the need to study the effects of neutron radiation.

Oilfield logging workers are a group frequently exposed to ^241^Am-Be neutron sources over long periods time. The human body is irradiated by the ^241^Am-Be neutron source through external exposure produced by fast neutrons and γ rays. Since the human body consists mainly of hydrogen, carbon, nitrogen, and oxygen, fast neutrons collide with hydrogen atoms in human tissues by elastic scattering to produce recoil protons and accompanying γ-rays. The recoil protons and γphotons lose energy by ionization and excitation in human tissues until all energy is absorbed. Additionally, 3 alpha particles can be produced in neutron-carbon nucleus interactions and 4 alpha particles can be produced in neutron-oxygen nucleus interactions. These alpha particles possess strong ionizing power and can damage sensitive living cells and tissues. ICRP publication 92 indicates that vast majority of neutrons produced by ^241^Am-Be are neutrons with 4 MeV energy, and their relative biological effect is 10, meaning their damage capacity is equivalent to 10 times the dose of γ-rays. This highlights the need for further studies on the multiple radiobiological indicators of neutrons (Neary et al., 1959; Broerse et al., 1968; Information needed to make radiation, 2006; Durante, 2014; Little, 2018; Wang et al., 2019; Holden et al., 2021; Wang et al., 2021). A wealth of knowledge has been accumulated regarding the radiobiology of neutrons. Various biological endpoint indicators have been studied *in vitro* cellular systems, including cell death, pathogenesis, cytogenetic damage, genomic instability, gene expression, mutation, and apoptosis (Neary et al., 1959; Broerse et al., 1968; Information needed to make radiation, 2006; Durante, 2014; Little, 2018; Wang et al., 2019; Holden et al., 2021; Wang et al., 2021), all supporting the notion damage is dependent on the radiation dose. It is worth noting that the mixed field of neutrons and γ produced by the ^241^Am-Be source may be more detrimental than single radiation. Research on the carcinogenic effects of neutrons has demonstrated that mixed-field radiation involving neutrons synergistically enhances the stochastic effects of carcinogenesis (Stichelbaut et al., 2014; Moriyama et al., 2019).

Radiobiological laboratory studies often focus on DNA damage, including chromosomal aberrations, micronucleus formation, genomic instability, and targeted gene expression. However, these effects are difficult to observe at long-term low doses(<20 mSv/year, ICRP Publication 60) and appear to be influenced by genetic, epigenetic and environmental factors, suggesting that the response is not exclusively dose-dependent (Broustas et al., 2017a). In the last two decades, there has been a shift in radiation biology from a DNA damage-centred paradigm to a non-target effect, where radiation-induced damage may be related to regulatory processes other than the target gene. Identifying a technique to reveal the radiation-specific response pathway may help to narrow down the study of genes and regulatory genes to some extent. Open chromatin is active chromatin and ATAC-seq can map this on a genome-wide level, providing distinct peaks representing specific active loci. To analyse gene expression differences in lymphocytes from oilfield workers exposed to neutron-γ radiation, we applied ATAC-seq and RNA-seq. The ultimate goal was to identify relevant pathways involving novel genes and transcriptional regulatory elements that are sensitive to low-dose neutron-γ radiation at the gene level.

## 2 Material and methods

### 2.1 Preparation of human lymphocytes

#### 2.1.1 Sample settings

The sample population comprised 451 healthy male workers who operated a ^241^Am/Be radioactive source and 73 healthy male workers from the same company who had never been exposed to a radioactive source. Exposure dose and health condition were evaluated to emphasize the need for sequencing (Tables 1, 2).

**TABLE 1:** Comparison of exposure dose between oil logging workers and other workers in the same company in 2022.

	Workers away from well logging source	Oil logging workers
Radiation resource	Cosmic rays, terrestrial rays, medical imaging	Cosmic rays, terrestrial rays, medical imaging and radioactive source used for logging
Radiation style	α,β,γ,X	α,β,γ,X and neutron (neutron dose rate: 450 μSv/h)
irradiated dose	2.4 mSv/year	3–53 mSv/year^a^

^a^ Dse from neutrons is about 0.48–46.48 mSv/year.

**TABLE 2:** Some data from the health examination of oil logging workers and other workers in the same company in 2022.

	Number of samples	Peripheral blood lymphocyte aberration rate(%)	Ocular lens opacity(%)	Prevalence of hypertension(%)
Oil logging workers	451	1.6	10	30
Workers away from well logging source	73	0	4	15

To form an radiation group and a control group in a 1:1 ratio, four workers with 5–30 years of radioactive service and four workers from the same company who had never been exposed to radioactive sources were selected. Members of the control group were labeled Wo1-Wo4, and members of the radiation group were labeled Wo5-Wo8. Workers in these two groups are paired one-to-one, with paired workers sharing the same age, area of residence, and daily commute, educational background, no smoking or drinking habits, and no recent history of antibiotic administration or medical radiation.

In order to increase the representativeness of the sample to the general population, 6 active radiological workers and 4 non-radiological workers were selected from the sample group again in the second stage. The two groups had the same average age and other admission criteria.

#### 2.1.2 Cell extraction and sequencing rules

Peripheral venous blood was obtained from the aforementioned members and collected in tubes containing anticoagulant EDTA. Immediately after blood collection, lymphocytes were extracted using the Percoll solution, with half of the cells used for the ATAC-seq program and the other half used for the RNA-seq program. The samples obtained at the two stages were separated for sequencing, and the results were combined for analysis. When the results are analyzed, the different parts of the sequencing results of the samples within the group are filtered out, keeping only the common parts to ignore individual differences among multiple samples within the group. Prior to sample collection, properly executed, written, and approved informed consent was obtained from each member.

### 2.2 RNA-seq (see the supplementary document for detailed message)

RNA degradation and contamination was monitored on 1% agarose gels. RNA purity, concentration, integrity was checked using Kits from US companies (more message in Supplementary document). A total amount of 1 µg RNA per sample was used as input material for the RNA sample preparations. Sequencing libraries were generated using NEBNext^®^ Ultra™ RNA Library Prep Kit for Illumina^®^ (NEB, United States) following manufacturer’s recommendations and index codes were added to attribute sequences to each sample. The clustering of the index-coded samples was performed on a cBot Cluster Generation System, After cluster generation, the library preparations were sequenced on an Illumina Hiseq platform and 125 bp/150 bp paired-end reads were generated. Criteria for determining differential genes: absolute value of foldchange greater than or equal to 2, and padjusted <0.05.

### 2.3 ATAC-seq (see the supplementary document for detailed message)

Before all steps, Cell acitivity was detected with Trypan blue assay and counted. ATAC-seq was performed as previously reported (Buenrostro et al., 2013). Briefly, nuclei was extracted from samples, and the nuclei pellet was resuspended in the Tn5 transposase reaction mix. The transposition reaction was incubated at 37°C for 30 min. Equimolar Adapter1 and Adatper 2 were added after transposition, PCR was then performed to amplify the library. After the PCR reaction, libraries were purified with the AMPure beads and library quality was assessed with Qubit. The clustering of the index-coded samples was performed on a cBot Cluster Generation System using TruSeq PE Cluster Kit v3-cBot-HS (Illumina) according to the manufactuer’s instructions. After cluster generation, the library preparations were sequenced on an Illumina Hiseq platform and 150 bp paired-end reads were generated. Nextera adaptor sequences were firstly trimmed from the reads using skewer (0.2.2). These reads were aligned to a reference genome using BWA, with standard parameters. These reads were then filtered for high quality (MAPQ ≥13), non mitochondrial chromosome, and properly paired reads (longer than 18 nt).

All peak calling was performed with macs2 using ‘macs2 callpeak --nomodel -- keepdup all --call-summits’. For simulations of peaks called per input read, aligned and de-duplicated BAM files were used without any additional filtering.

Criteria for determining differential peaks: absolute value of foldchange greater than or equal to 2, and padjusted <0.05.

### 2.4 GO and KEGG analysis

GO (http://www.geneontology.org/) is the International Standard Classification System for gene functions. It is a database established by the Gene Ontology Consortium, which covers GO Biological Processes, GO Cellular Components, and GO Molecular Functions. Genes or proteins can be identified by ID correspondence or by sequence annotation. Genes or proteins can be found by ID correspondence or sequence annotation, and the GO number can be used to correspond to Term, i.e., functional class or cellular localization. After screening for differential genes, enrichment analysis examines the distribution of differential genes in the Gene Ontology in order to elucidate the functional expression of the sample differences in the experiment. The software method we used for GO enrichment analysis is GOseq (Young et al., 2010), which is based on the Wallenius non-central hyper-geometric distribution, which is characterized by the difference in the probability of sampling an individual from within a category and the probability of sampling an individual from outside of a category. This distribution is characterized by the difference in the probability of drawing an individual from a category and the probability of drawing an individual from outside of a category, and this difference in probability is obtained by estimating the gene length preference, which allows us to more accurately calculate the probability that a GOterm is enriched by a differential gene.

KEGG (Kyoto Encyclopedia of Genes and Genomes) is a database for systematic analysis of gene functions and genomic information. As the main public database related to pathway (Kanehisa et al., 2007), KEGG provides excellent queries on integrated metabolic pathways, including metabolism of carbohydrates, nucleosides, amino acids, etc., and biodegradation of organic matter.The Pathway Significance Enrichment Analysis (PSE) is performed by applying hypergeometric test to find significant enrichment in differentially expressed genes compared to the whole genomic background, using the pathway as a unit in the KEGG database. The geometric test was applied to find out the pathways in which the differentially expressed genes were significantly enriched compared to the whole genomic background, and the *p*-value was calculated according to a specific formula, if *p* ≤ 0.05, it means that the differentially expressed genes were significantly enriched in that pathway. We used the software KOBAS (2.0) to perform the pathway enrichment analysis.

### 2.5 qPCR: confirming the effect of mixed neutron-gamma radiation on RPS18 and RPS27A expression

Three healthy volunteers were selected to provide about 50 mL of venous blood each, and each individual’s blood was irradiated individually to detect the expression of RPS18. Peripheral venous blood was collected using tubes containing the anticoagulant EDTA. Immediately after collection, lymphocytes were extracted using Ficoll-Paque solution. The extracted lymphocytes were then divided into seven groups and added to 1640 medium. These samples were carefully positioned around a radiation source on a specially designed rack to ensure uniform radiation. The temperature of the radiation chamber was set at 37°C to maintain optimal conditions.

The seven groups of lymphocytes were subjected to different levels of radiation: 0 μGy, 900 μGy, 1800 μGy, 3600 μGy, 6000 μGy, 15600 μGy, and 22000 μGy. Following radiation, the cells were concentrated through centrifugation (One-third of the cells were used for qPCR analysis, while the remaining cells were utilized for flow cytometry).

To induce erythrocyte lysis, 1 mL of 1×BD Pharm Lysel was added to each sample after centrifugation. The cells were then washed and lysed with Trizol. Subsequently, qPCR experiments were conducted to assess the relative expression of RPS18 and RPS27A, with β-actin serving as the internal reference gene. RPS18 primers are RPS18 - F: ATACAGCCAGGTCCTAGCCA, RPS18 - R: TTATTAACAGACAAGGCCTACAGA. RPS27A primers are RPS27A-F: TGTTGAGACTTCGTGGTGGT, RPS27A-R: AAACACCCCAGCACCACATT.

### 2.6 Protein-protein interaction (PPI)

The annotated differential gene names of the radiation group and the control group were entered into the STRING website, with gene admission criteria set as genes with a string score(confidence score) greater than 700 and a minimum required interaction score of 0.9. Then a protein-protein interaction (PI) network was produced.

### 2.7 Ribosomal protein detected by flow cytometry

Cell preparation was conducted according to step 5 of the protocol. As ribosomal proteins are located inside the cell, prior to staining, cell membrane punching was performed. Each cell sample was fixed with formaldehyde and treated with 1 mL of 1× BD Perm/Wash Buffer punching solution for 20 min, followed by thorough washing.

To facilitate staining, primary antibodies specific to RPS18 (Thermofisher#PA5-88211) and RPS27A (proteintech#14946-1-AP) were added to the cell samples, along with a BSA blocking solution. The samples were then incubated at room temperature for 2 h. After another round of washing, a secondary antibody labeled with FITC was added to the samples and incubated for an additional 2 h at room temperature.

Finally, flow cytometry analysis was performed using the BD FACSARIA Fusion instrument to detect and analyze the stained cells.

### 2.8 Detection of cell cycle and cell cycle-related proteins

#### 2.8.1 Cell cycle detection

Cell preparation was conducted according to step 5 of the protocol. The cells were fixed overnight with ice ethanol. Subsequently, the samples were washed to remove any residual ethanol. To assess the cell cycle, each sample was treated with 0.5 mL of PI/RNAse (Invitrogen#F10797) and stained at 4°C for 30 min, ensuring the absence of light exposure. The BD FACSARIA Fusion flow cytometer was employed to detect the cell cycle. The PE channel was selected to capture the PI signals during the detection process.

#### 2.8.2 Detection of cell cycle-related proteins

Cell preparation was performed according to step 5 of the protocol. To ensure proper staining of cyclin P21 and CDK2, cell membrane punching was conducted prior to the staining process. After formaldehyde fixation, each cell sample was treated with 1 mL of 1× BD Perm/Wash Buffer punching solution for 20 min, followed by thorough cleaning.

For staining, CDK2 monoclonal antibody (PE) and p21 monoclonal antibody (AF647) were added to each sample at recommended concentration. The samples were then incubated at 4°C for 30 min, while being shielded from light. Following the incubation, the samples were washed to remove any unbound antibodies. They were then suspended in PBS and immediately subjected to detection using the BD FACSARIA Fusion flow cytometer.

Antibodies for flow cytometry were purchased from CST (United States).

### 2.9 Statistical analysis

Statistics IBM SPSS 21.0 was used to perform the Student’s t-test to test differences in peak enrichment of TOP 10 transcription factors.

## 3 Results

### 3.1 Health risks in population sample

The types and doses of exposure experienced by the loggers were higher than those of other workers in the same company (Tables 1, 2). Furthermore, the maximum exposure dose exceeded the annual exposure dose limit of 20 mSv/year. As a result, the logging workers had a higher rate of chromosomal aberrations and a greater prevalence of lens opacity and hypertension compared to the control group.

### 3.2 RNA-seq: compared with the control group, differential genes were predominantly downregulated, and the genes in ribosome biogenesis was clearly clustered

**FIGURE 1:**
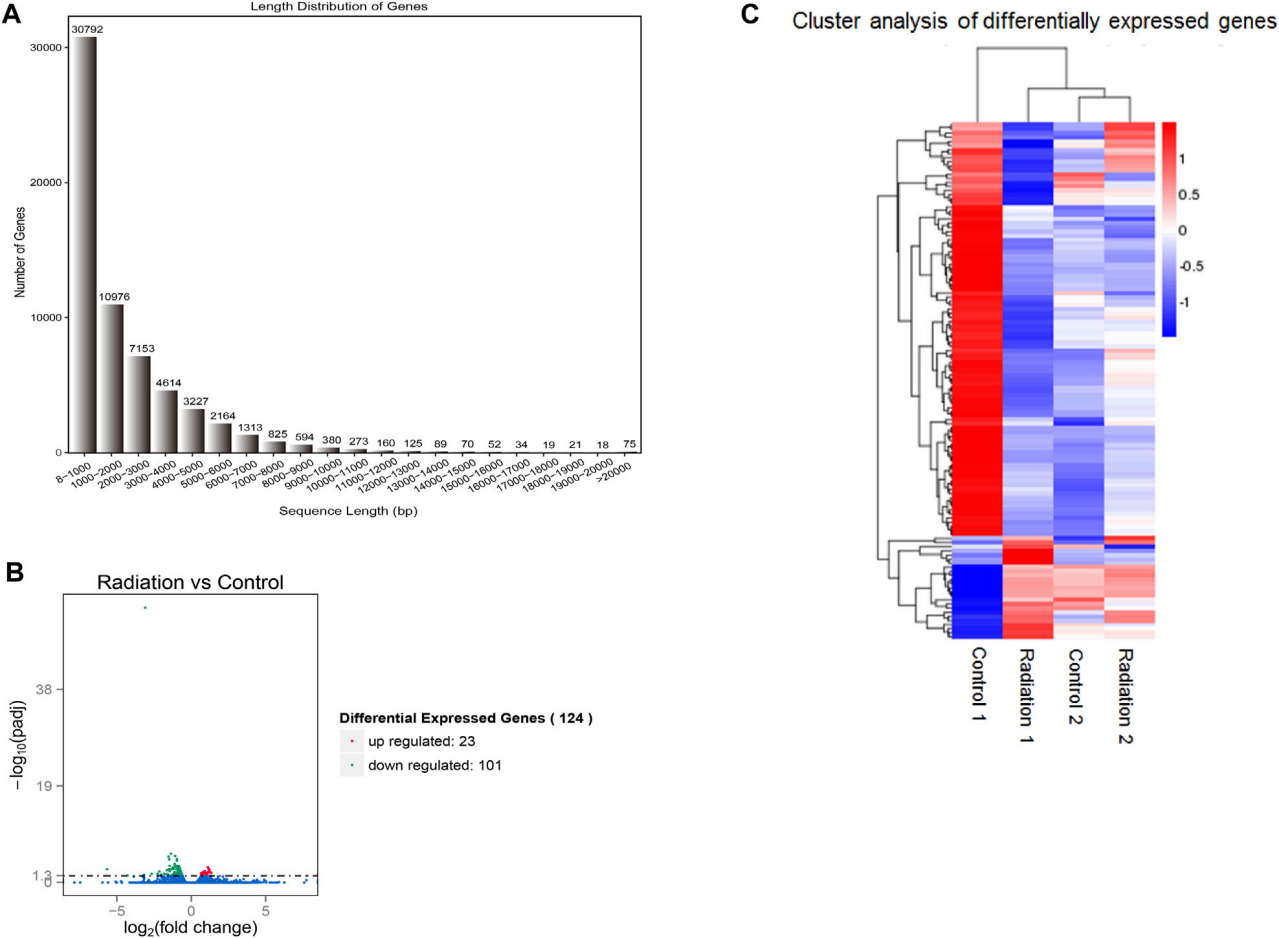
Visualization of gene length distribution and differential genes. **(A)** The X-axis showed the range of lengths of the transcript sequences, the Y-axis showed the numbers of unigenes. **(B)** The red dots signified genes that were significantly up-regulated, while the green dots indicated genes that were down-regulated. **(C)** The sample group names were displayed horizontally, with genes represented on the vertical axis. Red indicated up-regulation, blue indicated down-regulation, and color similarity reflected clustering degree. “Radiation 1 vs. Control 1” represented the sequencing result for the first blood sample, while “Radiation 2 vs. Control 2” represented the sequencing result for the second blood sample.

The quality of the reads obtained by sequencing is shown in Supplementary Table S1. The characteristics of the reads obtained from lymphocytes of each sample were basically the same. The number of clean reads reached nearly 99%, and the number of reads meeting the requirement of Q3 (99.9% accuracy of each base) reached more than 91%. The correlation coefficient *R* ^2^ of gene expression level among samples was close to 1 (Supplementary Figure S1A). The distribution of FPKM (Fragments Per Kilobase of exon model per Million mapped fragments) in all samples is almost the same (Supplementary Figure S1B), which shows that the process of processing samples has good stability, reliability, and reproducibility. The RNA-seq libraries of all samples had the expected fragment length (Figure 1A), most of which were small enough to ensure full and reliable sequencing. The mapping of Reads on the reference genome is shown in Supplementary Table S2.

In the radiation group, there were 101 downregulated genes and 23 upregulated genes in lymphocytes compared to the Control group (Figure 1B), leading to a significant difference in gene expression profiles, with downregulated genes dominating. The DAG (Directed Acyclic Graph) of differential genes in the radiation vs. control group showed a striking similarity between the DAG of all differential genes and the DAG of downregulated differential genes (Supplementary Figures S2, S3), indicating that the genes of lymphocytes in the radiation group were predominantly downregulated. The top 10 terms of GO enrichment analysis were as the main nodes of the DAG, with graph endpoints pointing to ribosomal subunits.

**FIGURE 2:**
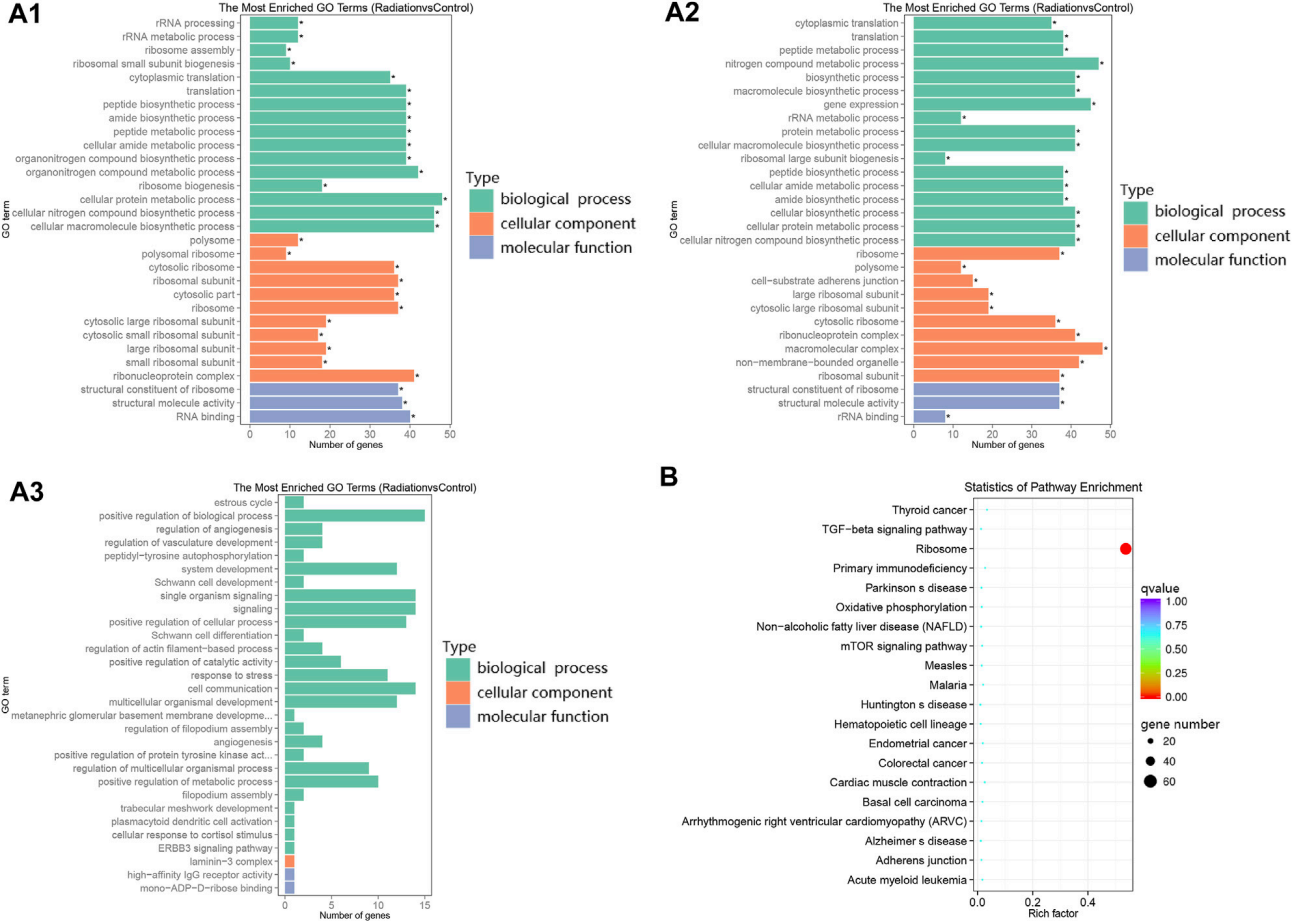
Clustering of differential genes in GO and KEGG. (2A1-2A3) The Y-axis represented the enriched GO terms, while the length of each bar represented the number of differentially expressed genes associated with that term. The different colors represented the three categories: biological process, cellular component, and molecular function. **(A1–A3)** represented the enriched GO terms for all differential genes, down-regulated differential genes, and up-regulated differential genes, respectively. **(B)** The Y-axis displayed the names of the pathways, while the X-axis represented the Rich factor. The size of the dots indicated the number of differentially expressed genes involved in each pathway, and the color of the dots corresponded to the different qvalue.

GO enrichment analysis revealed a limited number of enriched genes in upregulated terms, while the enrichment trend was pronounced in downregulated terms (Figure 1C; Figures 2A1-2A3). The main terms in biological processes were cytoplasmic translation, macromolecular synthesis, ribosome generation, and cellular protein metabolism; The cellular component was mainly concentrated in ribosome components; while molecular function was mainly concentrated in ribosome components, structural molecular activity, and rRNA binding. The enrichment analysis indicated a downregulation of ribosome production and a decline in anabolism. The only differential pathway for KEGG enrichment was the ribosome pathway, with 69 genes out of 134 background enriched in this pathway, all of which appeared in the descent pathway (Figure 2B; Table 3).

**TABLE 3:** Genes distributed on the ribosome term in KEGG.

Trem	Database	Input number	Background number	Corrected *p*-value	Gene IDs	Gene names
Ribosome	KEGG PATHWAY	69	134	4.24E-115	ENSG00000223803 ENSG00000226360 ENSG00000214389 ENSG00000182899 ENSG00000083845 ENSG00000183298 ENSG00000116251 ENSG00000197756 ENSG00000235174 ENSG00000236552 ENSG00000129824 ENSG00000234797 ENSG00000226221 ENSG00000214485 ENSG00000234287 ENSG00000230202 ENSG00000240509 ENSG00000231500 ENSG00000144713 ENSG00000234268 ENSG00000171858 ENSG00000216866 ENSG00000236801 ENSG00000244716 ENSG00000164587 ENSG00000235552 ENSG00000177954 ENSG00000112306 ENSG00000213178 ENSG00000147403 ENSG00000198242 ENSG00000136149 ENSG00000213860 NSG00000145592 ENSG00000137970 NSG00000226084 ENSG00000236439 NSG00000147604 ENSG00000205871 NSG00000228929 ENSG00000234742 NSG00000213862 ENSG00000220842 NSG00000226525 ENSG00000008988 NSG00000224631 ENSG00000229638 NSG00000131469 ENSG00000138326 NSG00000244363 ENSG00000237550 NSG00000185641 ENSG00000142937 NSG00000243199 ENSG00000139239 NSG00000143947 ENSG00000149806 NSG00000145425 ENSG00000109475 NSG00000212664 ENSG00000168028 NSG00000229119 ENSG00000232573 NSG00000071082 ENSG00000232346 NSG00000142541 ENSG00000156482 NSG00000198918 ENSG00000224094	RPS20P14 RPL10AP6 RPS3AP26 RPL35A RPS5 RPSAP19 RPL22 RPL37A RPL39P3 RPL13AP5 PS4Y1 RPS3AP6 RPL26P19 RPL7P1 - - RPL34P18 RPS18 RPL32 - RPS21 PS2P55 RPL24P8 - RPS14 RPL6P27 RPS27 RPS12 PL22P1 RPL10 RPL23A L13AP25 RPL21P75 RPL37 RPL7P9 - - RPL7 RPS3AP47 PS13P2 - - PL21P16 RPS7P10 RPS20 S27AP16 RPL4P4 RPL27 RPS24 PL7P23 - - RPS8 - RPL14P1 PS27A FAU RPS3A PL34 - RPSA - RPL3P4 RPL31 - RPL13A RPL30 RPL39 RPS24P8

### 3.3 ATAC-seq: compared with the control group, the upregulated genes were dominant in the ribosomal pathway, and transcription-related genes and genes related to the identification of DNA binding sites were most active

In order to correlate finely mapped open chromatin regions with gene expression profiles, lymphocytes were collected from the same individuals at the same time for ATAC and RNA sequencing. The live cells were suspended and loaded into the Tn5 transposition enzyme sequencing program. The results showed that all samples had similar peak enrichment distributions (Supplementary Figures S4A, B), with all ATAC-seq libraries displaying the expected fragment length. Most fragments were small, representing open chromatin between nucleosomes, while the large fragments spanning nucleosomes gradually decreased (Supplementary Figure S5A). The majority of ATAC peaks were mapped within 0.25 kbp of the transcription start site (Supplementary Figure S4B; Supplementary Figure S5B), suggesting that promoters are located in accessible chromatin regions. Further analysis revealed that the peaks of each sample were predominantly concentrated in the promoter (Supplementary Figure S5C), indicating that human lymphocytes have active gene elements. Moreover, a significant number of peaks were also enriched in intron and distal intergenic regions, implying the presence of enhancers or silencers.

**FIGURE 3:**
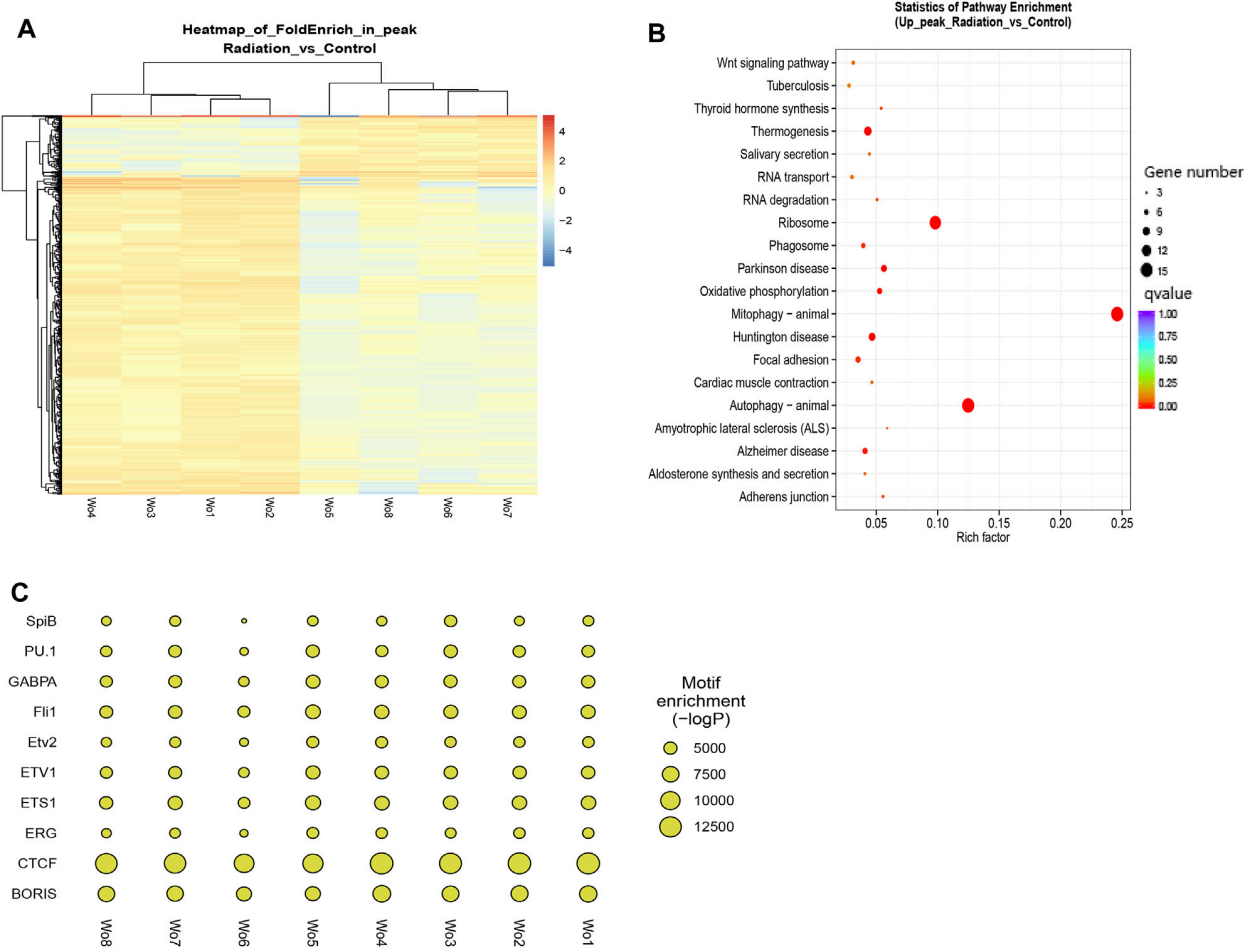
Genes were predominantly down-regulated, with the exception of the ribosomal pathway. **(A)** Each row represented a peak, and the columns represented the sample names. The color distinguished the similarity of fragment density on each peak, and clustering was performed based on the RPM value and FoldEnrich value of each peak. **(B)** KEGG enrichment in the down-regulated pathways was shown. The Y-axis displayed the pathway names, while the X-axis represented the Rich factor. The size of the dots indicated the number of differentially expressed genes associated with each pathway, and the color of the dots corresponded to the different qvalue. **(C)** The top ten enrichments of known transcription factor binding motifs in the two groups of samples were displayed. The size of the scatter plot represented the number of sequences with a particular motif.

**FIGURE 4:**
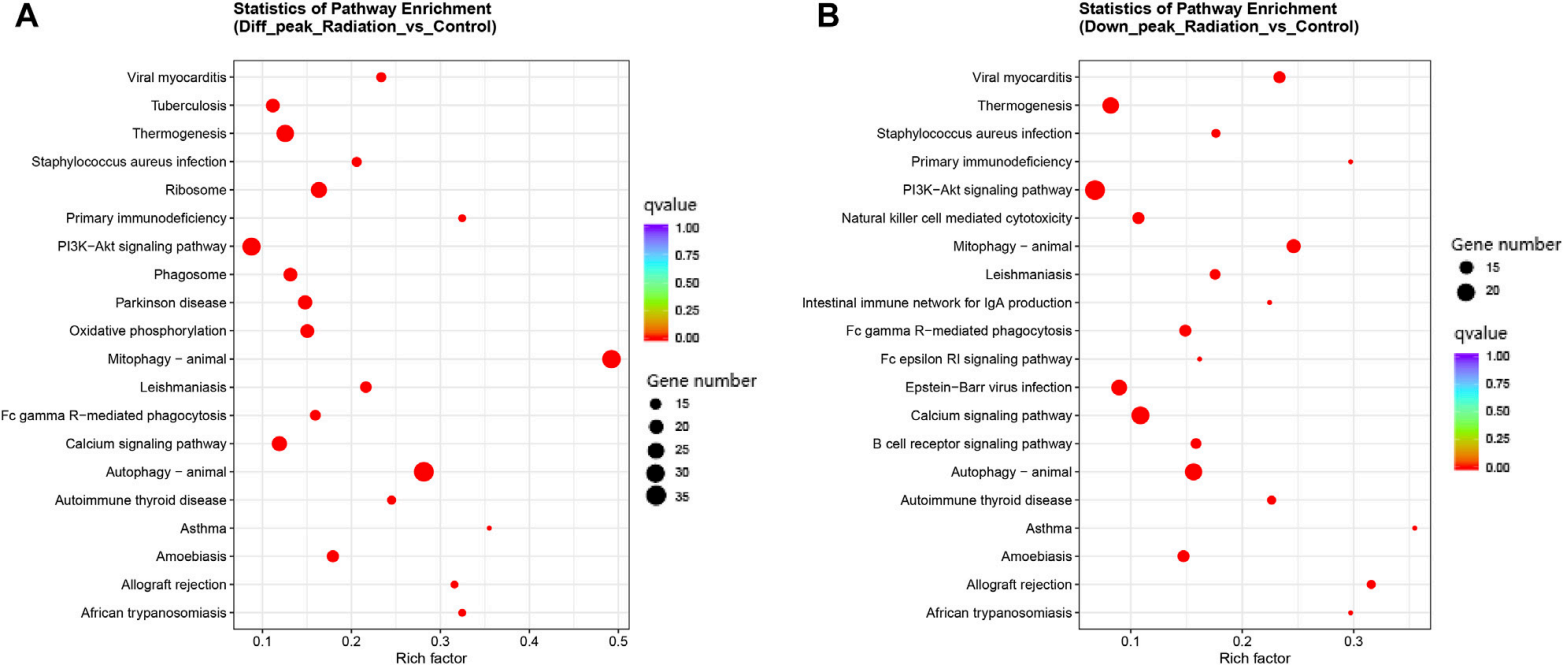
Clustering of all differential genes and down-regulated differential genes in KEGG in ATAC-seq. **(A, B)** KEGG enrichment in the total and up-regulated pathways. The Y-axis displayed the names of the pathways, while the X-axis represented the Rich factor. The size of the dots indicated the number of differentially expressed genes involved in each pathway, and the color of the dots corresponded to the different qvalue.

Differential peaks were clearly clustered, and genes corresponding to peaks obtained by ATAC-seq are concentrated in mitophogy-animal, autophage-animal, ribosome and some unexplained immune-related pathways. Both upregulated and downregulated genes are enriched in mitophogy-animal and autophage-animal pathways, while only upregulated genes are enriched in ribosome pathway (Figures 3A, B; Figures 4A, B). By listing the top 10 enriched pathways, it was observed that, in addition to the aforementioned three pathways, thermogenesis, calcium signaling pathway, and oxidative phosphorylation were also significantly at the forefront of pathway enrichment (Table 4).

**TABLE 4:** Top 10 KEGG pathways in ATAC.

Top terms	Corrected *p*-value	Top terms (down)	Corrected *p*-value	Top terms (up)	Corrected *p*-value
Mitophagy - animal	1.04E-27	Mitophagy—animal	1.77E-12	Mitophagy—animal	1.04E-16
Autophagy - animal	1.14E-24	Autophagy—animal	1.77E-12	Autophagy—animal	7.19E-13
Ribosome	2.78E-12	Viral myocarditis	9.52E-11	Ribosome	8.59E-11
Thermogenesis	8.28E-12	Calcium signaling pathway	1.16E-10	Thermogenesis	0.000448373
Parkinson disease	1.02E-09	Allograft rejection	1.16E-10	Parkinson disease	0.000469189
Leishmaniasis	1.54E-09	Asthma	2.83E-10	Huntington disease	0.00047664
Oxidative phosphorylation	1.68E-09	Primary immunodeficiency	1.04E-09	Oxidative phosphorylation	0.001775777
PI3K-Akt signaling pathway	3.08E-09	African trypanosomiasis	1.04E-09	Alzheimer disease	0.006875403
Calcium signaling pathway	3.08E-09	Autoimmune thyroid disease	1.79E-09	Focal adhesion	0.014624872
Amoebiasis	3.08E-09	Leishmaniasis	4.13E-09	Phagosome	0.017866394

The binding of transcription factors and positive regulatory sequences upstream of the promoter serves as a signal for gene activation. In our study, we tried to identify the binding sequences of known transcription factors within the TOP 10 peak enrichment, and compare the enrichment differences between the radiation and control groups (Figure 3C). Our results revealed that the binding sequences of transcription factors in the radiation group were less enriched than those in the control group. We listed the specific enrichment counts of the two groups and tested their mean values (Table 5). The findings were consistent, except for PU.1 (*p* > 0.05), where the enrichment of binding motifs of other transcription factors decreased in the radiation group with statistical significance. We further described the function of these transcription factors (Supplementary Table S3), where the most enriched CTCF, BORIS, and ETS1 were mainly involved in transcriptional regulation and identifying DNA binding sites.

**TABLE 5:** The average of Top ten enrichment of known transcription factor binding motifs in irradiated and control groups.

Transcription factor	Motif	Number of sequences with motif in radiation team^a^	Number of sequences with motif in control team^a^	*p*
SpiB	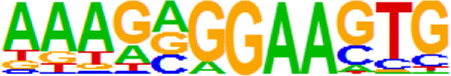	3417.25	3858.75	0.06
PU.1	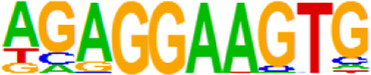	4236.75	4729.25	0.14
GABPA	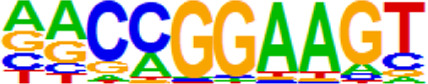	4642.5	4988.25	0.04
Fli1	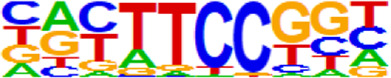	5151.75	5612.75	0.09
Etv2	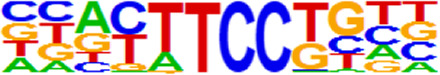	3782.5	4167.5	0.06
ETV1	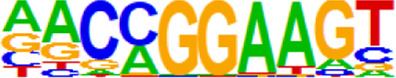	4688	5187.75	0.03
ETS1	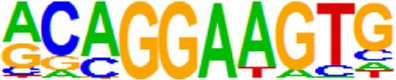	5342	5893.75	0.03
ERG	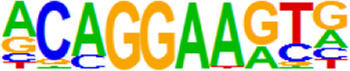	3607.5	4030.75	0.06
CTCF	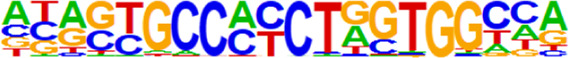	11462.5	13427.5	0.01
BORIS	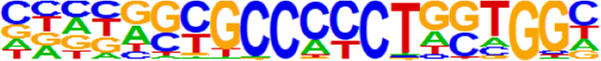	6808.75	7999.25	0.01

^a^ The average number of the samples in the group.

*p <* 0.05 means significant difference between the two groups.

### 3.4 Integration of RNA-seq and ATAC-seq analysis: compared with the control group, the genes in both RNA-seq and ATAC-seq showed a decreasing trend, and the expression trend of pseudogenes and their parent genes in the ribosomal pathway was opposite

**FIGURE 5:**
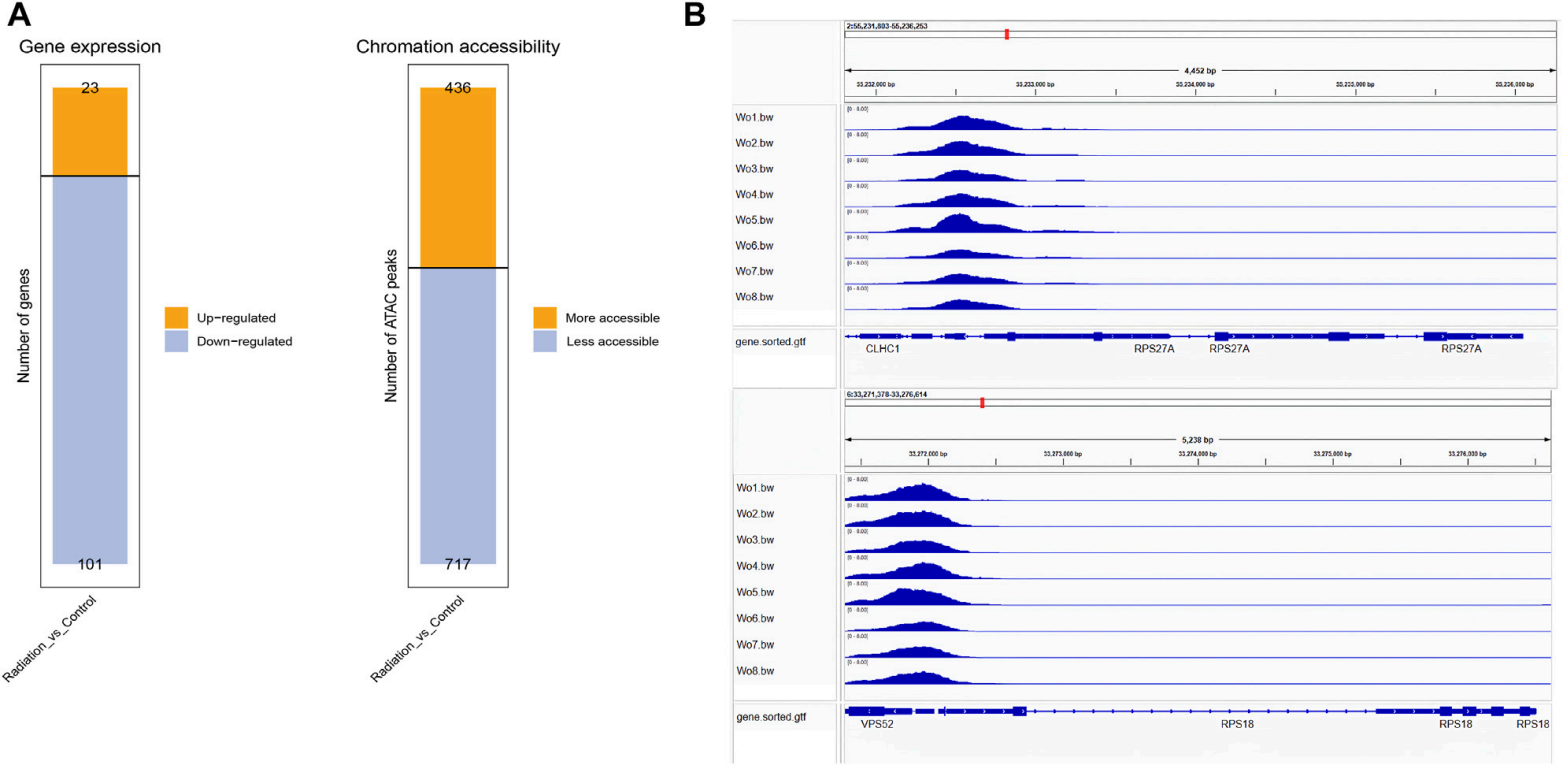
The tendency of downregulation was predominant in RNA-seq and ATAC-seq. **(A)** The figure displayed the up-regulated and down-regulated genes identified in RNA-seq, as well as the increased and decreased chromatin opening peaks observed in ATAC-seq. **(B)** The peak enrichment analysis of RPS18 and RPS27A in the ATAC sequencing library revealed that the enrichment peak was lower in the irradiated group (WO5-WO6, except Wo5) compared to the control group (Wo1-Wo4).

**FIGURE 6:**
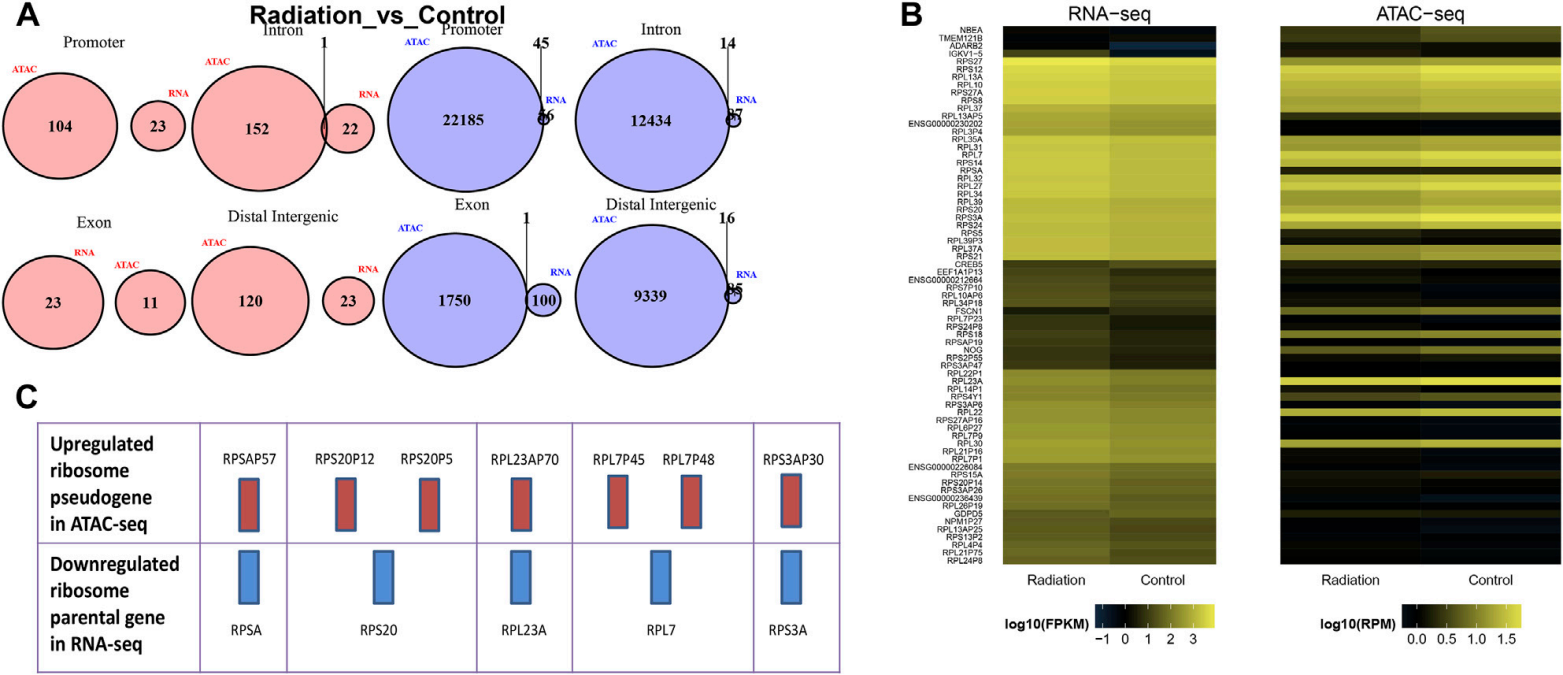
Comparison of results of RNA-seq and ATAC-seq. **(A)** The figure illustrated the differential peak-related genes in ATAC, which were categorized into four regions: Promoter, Intron, Exon, and Distal Intergenic region. These regions were intersected with the up-regulated and down-regulated genes identified in RNA-seq. Pink and blue colors indicated up-regulation and down-regulation, respectively. **(B)** The figure compared the fragment density between the two sequencing programs for two groups of samples. Each row represented a gene or a peak, and the color indicated the distribution density of fragments. The color change trend indicated the direction of up-regulation or down-regulation. **(C)** In the same column, the red bar represented up-regulated genes in ATAC-seq, while the blue bar represented down-regulated parental genes in RNA-seq.

To gain deeper insights into the effects of neutron-γ radiation on DNA or RNA within the gene expression pathway, we employed a combined analysis of ATAC and RNA sequencing. Our aim was to identify more specific and unique response mechanisms. The results of ATAC-seq and RNA-seq analysis revealed significant differences in gene expression between the exposed lymphocytes and the group, with a dominant trend of downregulation (Figure 5A). Specifically, RNA sequencing identified 23 upregulated and 101 downregulated genes, while ATAC-seq sequencing revealed 434 upregulated peaks and 717 downregulated peaks. To confirm if the same genes are regulated in the same direction in both ATAC-seq and RNA-seq, we selected RPS18 and RPS27A from the genes that showed significant downregulation in RNA-seq. We then performed tracks analysis on the entire sequences of these two genes based on the ATAC-seq library information (Figure 5B). Interestingly, we found that the region of the upstream peak enrichment was the same in all samples. As expected, the enrichment peak height of the radiation group (WO6-WO8) was lower than that of the control group (Wo1-Wo4). Notably, the chromatin openness and gene alteration trends were consistent for both genes. Our primary focus was on the overlap between the two sequencing results. The venn diagram and heat map (Figures 6A, B) indicated the presence of overlapped genes between ATAC-seq and RNA-seq in both the radiation and control groups, with more overlap observed in the downregulated genes. However, the two directions were not always the same, and even the genes in the two sequences did not show significant intersection in the KEGG enrichment. We only analyzed the list of different genes in the ribosome pathway of interest and found that most of the genes significantly upregulated in ATAC-seq in the ribosome pathway were pseudogenes. Interestingly, transcripts of the parent genes of some pseudogenes were present in significantly downregulated gene sets in RNA-seq (Figure 6C; Table 6; Supplementary Table S4).

**TABLE 6:** DEG upregulated in ATAC-seq and downregulated in RNA-seq in the ribosome pathway.

Seq-type	Gene ID associated with the ribosome pathway
ATAC	ENSG00000240366|ENSG00000232228|ENSG00000241052|ENSG00000275160|ENSG00000225893|ENSG00000236425|ENSG00000230198|ENSG00000236888|ENSG00000224646|ENSG00000239201|ENSG00000229554|ENSG00000228599|ENSG00000270764|ENSG00000187504|ENSG00000237409|ENSG00000225661|ENSG00000237784|ENSG00000244422|ENSG00000267524|ENSG00000228601|ENSG00000276763|ENSG00000225823|ENSG00000169253|ENSG00000228744|ENSG00000260192
RNA	ENSG00000223803|ENSG00000226360|ENSG00000214389|ENSG00000182899|ENSG00000083845|ENSG00000183298|ENSG00000116251|ENSG00000197756|ENSG00000235174|ENSG00000236552|ENSG00000129824|ENSG00000234797|ENSG00000226221|ENSG00000214485|ENSG00000234287|ENSG00000230202|ENSG00000240509|ENSG00000164587|ENSG00000144713|ENSG00000234268|ENSG00000171858|ENSG00000216866|ENSG00000236801|ENSG00000244716|ENSG00000231500|ENSG00000235552|ENSG00000177954|ENSG00000112306|ENSG00000213178|ENSG00000147403|ENSG00000198242|ENSG00000136149|ENSG00000213860|ENSG00000145592|ENSG00000137970|ENSG00000226084|ENSG00000236439|ENSG00000147604|ENSG00000205871|ENSG00000228929|ENSG00000234742|ENSG00000213862|ENSG00000220842|ENSG00000226525|ENSG00000008988|ENSG00000224631|ENSG00000229638|ENSG00000131469|ENSG00000138326|ENSG00000244363|ENSG00000237550|ENSG00000185641|ENSG00000142937|ENSG00000243199|ENSG00000139239|ENSG00000143947|ENSG00000149806|ENSG00000145425|ENSG00000109475|ENSG00000212664|ENSG00000168028|ENSG00000229119|ENSG00000232573|ENSG00000071082|ENSG00000232346|ENSG00000142541|ENSG00000156482|ENSG00000198918|ENSG00000224094

### 3.5 Downregulation of RNA expression of RPS18 and RPS27A in lymphocytes after neutron gamma radiation

**FIGURE 7:**
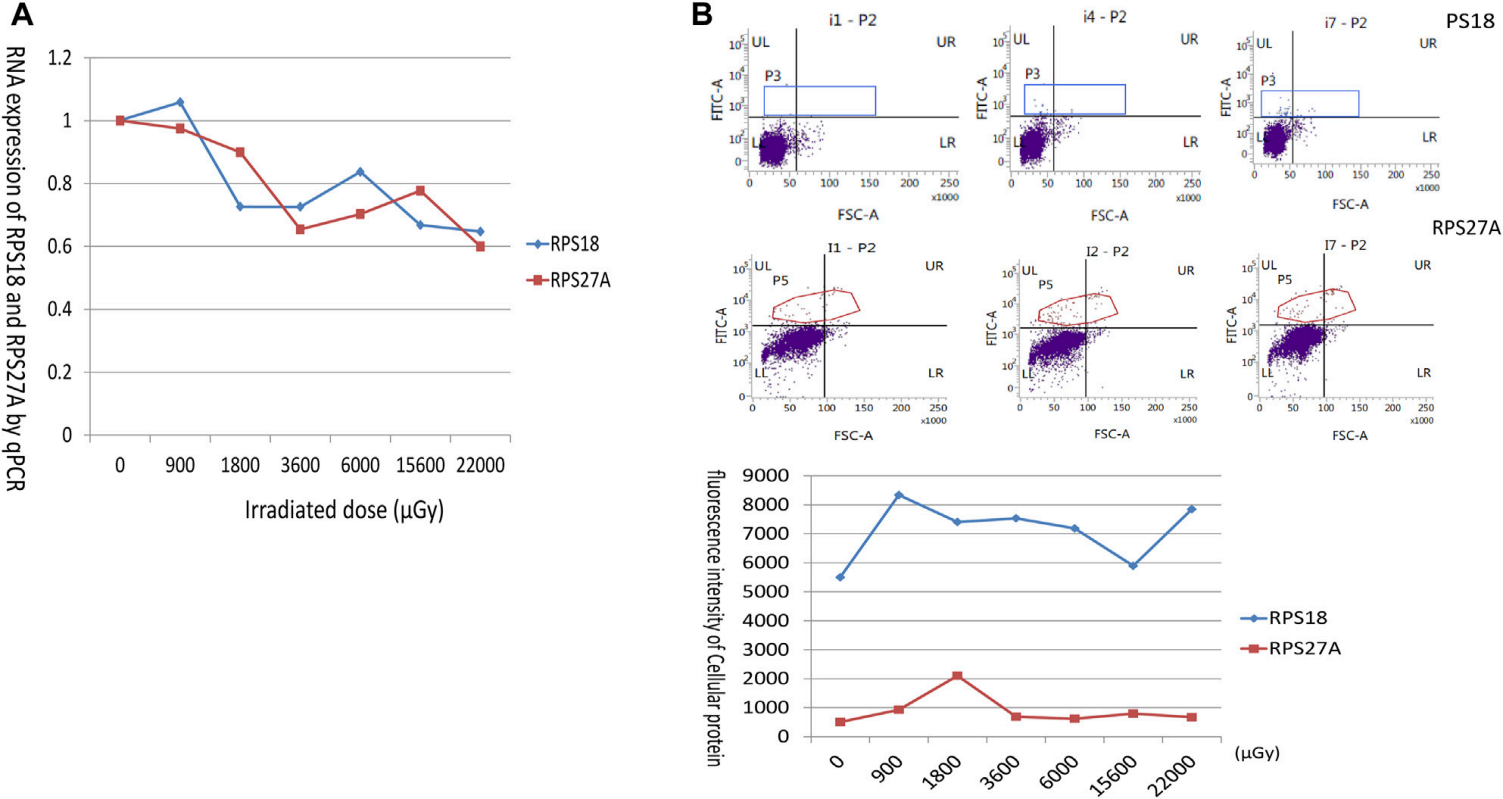
Trends in mRNA and protein expression of RPS18 and RPS27A with exposure dose. **(A)** The horizontal coordinate showed the irradiated dose, and the vertical coordinate showed the mRNA expression of RPS18 and RPS27A relative to β-actin. The expression of RPS18 and RPS27A decreased with increasing irradiated dose. **(B)** The flow cytometry analysis of RPS18 and RPS27A protein expression. RPS18 protein levels remained high following irradiation, prompting the selection of i1 (0 μGy) pre-exposure, i4 (3600 μGy) intermediate dose exposure, and i7 (22,000 μGy) maximum dose exposure for presentation. Conversely, RPS27A expression exhibited an initial surge upon irradiation initiation, followed by a subsequent decline. Therefore, i1 (0 μGy) pre-irradiation, i2 (900 μGy) initial low-dose irradiation, and i7 (22,000 μGy) maximum-dose irradiation were chosen for display. The graph at the bottom delineated the dose-dependent alterations in RPS18 and RPS27A protein expression.

To confirm the downregulation of RNA expression of ribosomal proteins (RPs) in the sequencing results, the mRNA expression of RPS18 and RPS27A was selected as indicators to analyze their response to the radiation source. The results showed that the RNA expression of RPS18 and RPS27A in unirradiated lymphocytes was almost the same as that of the internal reference gene β-actin. The relative RNA expression of RPS18 decreased rapidly to below 0.8 with increasing radiation doses, accompanied by slight fluctuations in the overall slow decrease, and the change of RPS27A was similar to that of RPS18 (Figure 7A).

### 3.6 PPI: the main functions of the proteins corresponding to the differential genes are ribosome biogenesis, RNA binding, and translation

**FIGURE 8:**
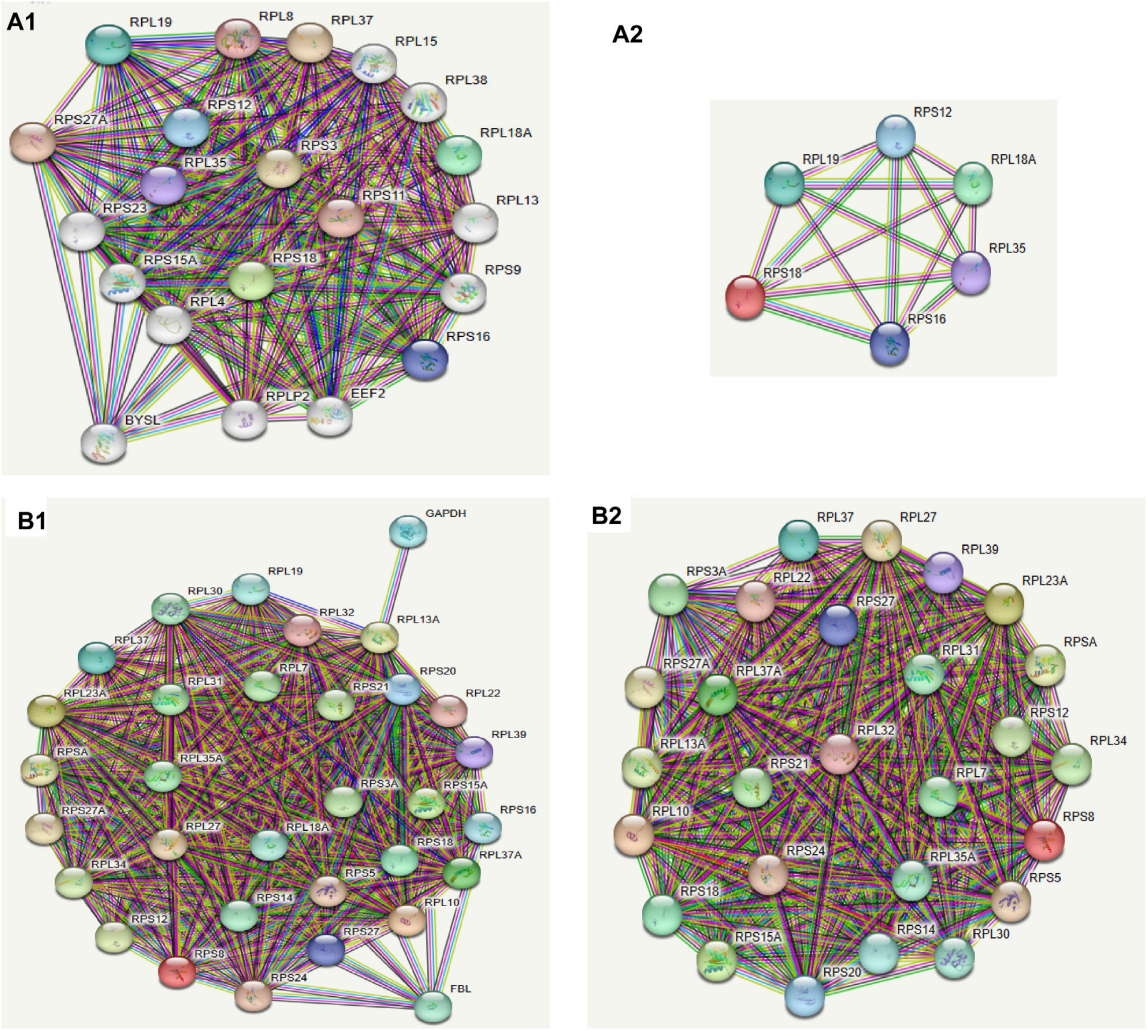
Ribosomal protein were very important in protein-protein interaction networks. **(A1)** Protein-Protein Interaction Networks of differentially expressed genes. The colors of the circles represented similar functions or intracellular adjacencies. **(A2)** The 6 most related gene proteins, all of which are ribosomal proteins. **(B1)** Protein network interactions of differentially expressed genes in the ribosome pathway. The colors of the circles represented similar functions or intracellular adjacencies. **(B2)** The most related gene proteins were also ribosomal proteins in the ribosome pathway.

The ribosomal pathway genes were found to be widely downregulated in both ATAC-seq and RNA-seq. To explore the relationship between the corresponding proteins, we employed the STRING platform to analyze the protein-protein interaction networks (PPI) for all differential genes and differential genes (fold enrichment >2 or <2, qpvalue<0.05) in the ribosomal pathway based on the access conditions, respectively. Using different colors to identify clusters, we observed that almost all the closely related genes corresponded to ribosomal proteins, with the proteins with the strongest correlation being ribosomal proteins(Figure 8). The report of these two analyses revealed that the clustering of molecular components, molecular function, and biological processes was similar. Cellular components was clustered in the cytosolic small ribosomal subunit, cytosolic ribosome, and cytosolic large ribosomal subunit, molecular function was mainly clustered in structural constituent of ribosome, rRNA binding, and RNA binding, while biological processes were mainly clustered in SRP-dependent cotranslational protein targeting to membrane, nuclear-transcribed mRNA catabolic process, nonsense-mediated decay, translational initiation, and cytoplasmic translation.

### 3.7 Expression of the ribosomal proteins RPS18 and RPS27A

Both RPS18 and RPS27A exhibited abundant expression levels in all lymphocytes. Following radiation, RPS18 displayed a rapid increase in expression, when the dose reached 900 μGy. Further increases in dose did not result in additional elevation, as RPS18 remained consistently high. On the other hand, RPS27A also exhibited a rapid doubling of expression in response to early exposure, mirroring the behavior of RPS18. However, it appeared to be influenced by additional factors, leading to a subsequent decline in expression levels. Nevertheless, RPS27A still maintained slightly higher levels compared to the pre-exposure state (Figure 7B).

### 3.8 Results of cell cycle assays

**FIGURE 9:**
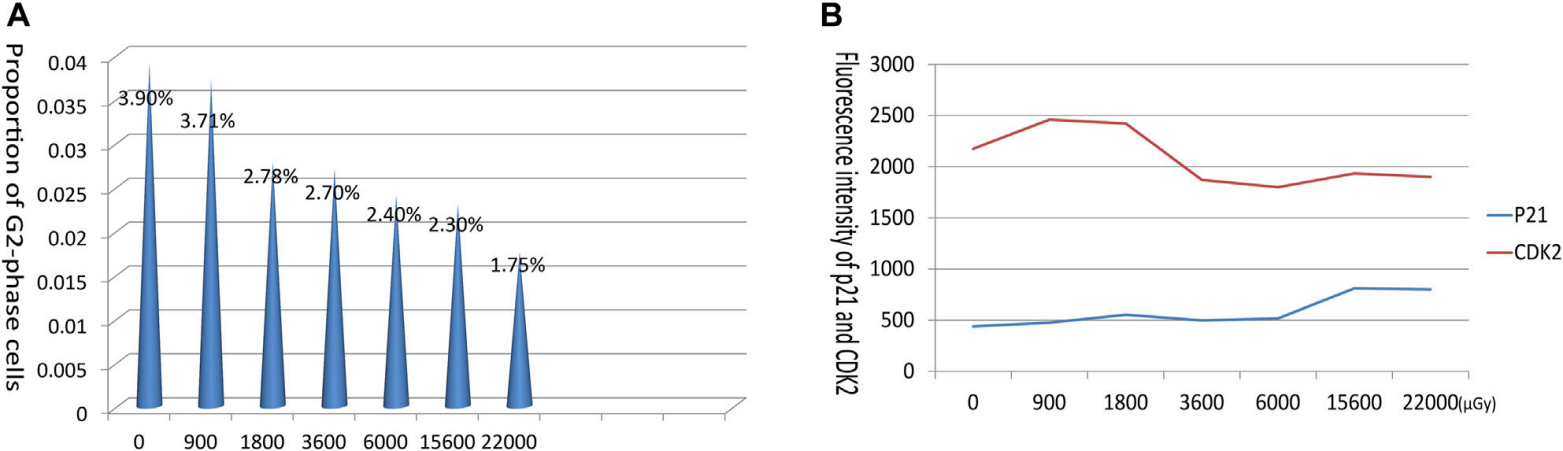
Alterations in cell cycle distribution and expression of cycle-related proteins in response to varying doses. **(A)** The proportion of cells in G2 phase decreased with increasing dose. **(B)** Line graph of cyclin P21 and CDK2 expression with dose changes.

Low-dose radiation seems to have minimal impact on the overall distribution of the cell cycle. In normal human lymphocytes, a significant proportion of cells reside in the G1 phase, representing the static phase. Detecting any discernible response to low-dose radiation in these cells proves challenging, as only a slight decrease in the proportion of cells in the G2 phase is observed. The expression of the cell cycle arrest-promoting protein, P21, exhibited a gradual increase following radiation. Conversely, the expression of CDK2, which facilitates the progression through cell cycle checkpoints, initially increased but subsequently returned to pre-radiation levels (Figure 9).

## 4 Discussion

The monitored exposure doses and occupational health results indicated that logging workers were exposed to higher levels compared to the control group. Moreover, their positive rates for some indicators of radiation damage exceeded those of non-radiological workers within the same company, suggesting that neutron-γ radiation may have an impact on workers’ health. While radiation may not be a significant factor it was found to have some effect on chromosomal alterations and potential diseases, emphasizing the importance of our sequencing efforts aimed at identifying genetic alterations.

Studies on the effects of neutron radiation on cells have traditionally focused on assessing cell viability, activity, proliferation, cell cycle, and chromosome morphology. However, the integration of sequencing technologies has significantly advanced the exploration of molecular pathways in recent years (Broustas et al., 2017a; Broustas et al., 2017b; Bouten et al., 2021; Shimabukuro et al., 2022). Mice exposed to high doses of neutrons are relatively simple animal models that can be used to study gene sequence and pathway changes in whole blood cells (Broustas et al., 2018), Some of the findings in our study are similar to those reported in previous studies, such as the identification of differential pathways associated with the inhibition of ribosome biogenesis. The discrepancy between the results of our study and previous neutron-induced genetic assays is likely due to differences in experimental conditions. Specifically, those prior experiments utilized artificially high doses of radiation on cultured cells for short periods of time, leading to divergent outcomes (Broustas et al., 2017a; Bouten et al., 2021; Grandt et al., 2023). Nevertheless, the effects of low doses of neutron-γ radiation on genes remain unexplored. In particular, the alteration of gene expression in human *in vivo* cells following long-term radiation is a novel aspect of our study. Radiation’s primary impact is on the unwinding of DNA (Tsoulou et al., 2005), making the detection of open regions of chromatin the more suitable method. This is precisely what ATAC-seq can address.

ATAC-seq provides significantly higher signal-to-noise ratios and more precise mapping of open chromatin regions than other methods (Tsompana and Buck, 2014). By integrating ATAC-seq and RNA-seq data, we were able to more accurately identify differential genes and their corresponding upstream and downstream regulatory elements. To ensure the reliability of our sequencing results, we extracted a single type of radiation-sensitive lymphocytes and thoroughly examined quality reports, with a particular attention on sample reproducibility (Supplementary Figures S1A, B; Supplementary Figures S4A, B, 5B). We confirmed that the distribution of sequenced fragments was highly stable, and that our correlation tests met all quality control requirements. To enhance the reliability of our positive rate calculations, we filtered out any divergent results among samples within each group. We only retained the intersectional portion of samples within each group before analyzing differences between groups. To maintain *in vivo* cellular characteristics, we extracted and froze lymphocytes immediately after venous blood collection. This approach ensured that any genetic interference after leaving the organism was avoided.

The visualised results of RNA-seq (Figures 1B, C; Supplementary Figures S2, S3) showed a clear difference in expression between the radiation and control groups. The differences in biological processes were mainly in protein metabolic process, protein translation and biosynthesis. For cellular components, the differences were mainly in ribosome synthesis. The differences in molecular function were mainly in ribosome structural components, structural molecular activity and RNA binding. Apparently, the observed differences were primarily clustered within the translational synthesis program, which included RNA binding and ribosome composition. In contrast to the changes in the transcriptional profile of CD4^+^ T cells induced by pure low-dose γ-photons, which were predominantly associated with mitochondria (Cho et al., 2018), neutron-γ radiation primarily affected ribosomes, underscoring the very different biological mechanisms induced by different types of radiation. Perhaps the biological effects induced by neutron radiation at different doses or dose rates are also distinct. Broustas CG et al. Conducted microarray sequencing after subjecting human whole blood to high-dose rapid radiation, revealing 125 differentially expressed genes (Broustas et al., 2017a). Some of these genes are associated with the cell cycle, which is related to the need for repair in acutely damaged cells for survival (Bouten et al., 2021). This difference is attributed to the distinct modes of neutron radiation and also represents a mixed result of different cell types in whole blood. In our experiment, we examined workers exposed to low-dose long-term radiation, and the genes that are preferentially responsive is inevitably different. Additionally, the sequenced cells were purified lymphocytes, which further facilitate the elucidation of specific responses to neutron radiation.

Upon scrutinizing the GO enrichment outcomes, we observed that the upregulated genes were comparatively less enriched. A more lucid representation of this phenomenon can be viewed in the DAG plot (Supplementary Figures S2, S3), which reveals that the functional orientation of all differential genes is essentially akin to that of the downregulated differential genes. This finding implies that the principal effect of low-dose neutron-γ radiation on gene regulation is gene downregulation, aligning with the trend of gene alteration of blood cells exposed to high-dose neutron-γ radiation (Broustas et al., 2018; Mukherjee et al., 2019), This effect is distinct from the regulation of genes by pure X-rays, and the underlying reasons for this difference remain unclear. It is possible that the differential damage caused by neutrons and photons contributes to this disparity. Notably, the KEGG enrichment analysis unequivocally indicated a singular differential pathway - the downregulated ribosome pathway, which corroborated the outcomes of the GO analysis and narrowed down the analysis to the ribosome pathway exclusively. Within the ribosome pathway, there were a total of 134 background genes, of which 69 were downregulated and enriched (Figure 2B; Table 3), emerging as the most prominent data in this experiment.

Consistent with our expectations, ATAT-seq yielded the same stable distribution of small fragments as observed in Supplementary Figure S5A. These fragments represent open chromatin regions located between nucleosomes and constitute the primary target for sequencing. Notably, an average of 57.81% of ATAC peaks were mapped within 300bp of promoter (Supplementary Figures S4A, B), indicating a significantly higher promoter openness in lymphocytes than most human cells. This observation highlighted the active regulation of genes in the open state of lymphocytes. Furthermore, the peak enrichment in intron and distal intergenic regions suggested that, in addition to promoters, there is extensive gene regulation. Ultimately, the intricate inter between promoters, enhancers, silencers, and trans-acting transcription factors determined mRNA expression.

We were interested in the distribution and clustering of differential peaks. To achieve this, we utilized heat maps for differential analysis, as depicted in Figure 3A. The maps clearly demonstrated that there was a distinct difference in chromatin distribution between the radiation group and the control group. By analyzing the pathway where peaks are located and their relationship, it is possible to uncover the regulatory mode of neutron-γ radiation at the DNA level. Our KEGG enrichment analysis of ATAC-seq revealed that the differential peaks were predominantly concentrated in pathways related to mitophagy-animal, autophagy-animal, and ribosome(Figure 3B; Figures 4A, B). Notably, both upregulated and downregulated genes were enriched in the mitophagy-animal and autophagy-animal pathways. Neutron-γ radiation appears to have a bidirectional regulation on mitosis and autophagy. Conversely, the ribosome pathway only exhibited enrichment of upregulated genes (Figure 3B). As previously discussed, the RNA-seq analysis revealed that only the ribosomal pathway was downregulated. Based on this finding, it is reasonable to hypothesize that the regulation of mitophagy and autophagy at the DNA level did not result in the expression of corresponding mRNAs. Conversely, the upregulation of the ribosomal pathway through opening chromatin ultimately led to a decrease in ribosome-associated transcription.

The KEGG enrichment analysis in ATAT-seq revealed that, in addition to the three mentioned pathways, thermogenesis and oxidative phosphorylation were also clearly among the top 10 enriched pathways, as shown in Table 4. The two pathways may play a role in regulating the open state of chromatin by influencing nucleosome movement through the regulation of ATP energy supply.

Enrichment of transcription factor binding regions (promoters) has been shown to be reliable predictors of gene expression intensity. To further investigate the activity of differential genes in their respective pathways, we performed a peak-enrichment analysis of the binding motifs of the top 10 known transcription factors. We queried the functions of these transcription factors in the National Library of Medicine (Supplementary Table S3) and found that they mainly activate gene expression in lymphocytes, promoting their proliferation and differentiation. These findings suggested that neutron-γ radiation may impede lymphocyte proliferation and differentiation by down-regulating transcription and inhibiting DNA recognition. For example, the proteins encoded by CTCF, BORIS, and ETS1 are mainly used for transcriptional regulation and DNA binding. The observed decrease in openness of DNA regions in the radiation group suggested a reduction in transcriptional recognition of DNA, resulting in a subsequent decrease in the expression of target genes. This might also explain the downregulation of ribosome-related genes, as previous studies have shown that knockdown of the transcription factor ETS1 gene led to a significant decrease in transcriptional activity of ribosome-associated genes (Xiao et al., 2022). Our tracks analysis of RPS18 and RPS27A revealed a decreased enrichment within 500 bp upstream of these genes in the irradiated cells, indicating that the decreased expression of these genes in the radiation group was likely due to a reduction in the enrichment of their binding regions for transcription factors.

To gain a more comprehensive understanding of the data, we examined the corresponding differential genes in both ATAC-seq and RNA-seq (Figure 5A; Figures 6A, B). In both sequencing methods, the majority of differential genes in the radiation group were downregulated, suggesting that neutron-γ radiation may lead to a decrease in the activity of these, in line with findings by Constantinos G(14). The heat map revealed some identical differential genes in both sequences, with most of these genes exhibiting the same directional regulation (color change). The tracks analysis of RPS18 and RPS27A further supported the consistency of the two sequencing results. Notably, in our experiment, the upregulation of the ribosome was dominant in ATAC-seq, while the downregulation of the ribosome pathway was dominant in RNA-seq. This discrepancy can be attributed to the fact that the upregulated genes of the ribosome pathway in ATAC-seq and the downregulated genes of the ribosome pathway in RNA-seq were not the same set of genes. In other words, our findings suggested that the upregulated genes of the ribosome pathway in ATAC-seq did not result in a corresponding upregulation of mRNA, while the downregulated genes of the ribosome pathway in RNA-seq were not significantly downregulated at the DNA level. This highlighted the fact that the regulation from DNA to RNA was not a straightforward linear process, but rather involved other regulatory elements and trans-acting factors that allow for the target gene to be expressed at a certain intensity. (Sekihara et al., 2018; Schmidt et al., 2023). It is important to note that our hypothesis based solely on sequencing results had some limitations. Highly ionizing radiation not only triggers genetic changes but also directly damages ribosome DNA. Extensive investigations and experiments have revealed that chronic neutron-γ radiation can cause cellular ribosomal DNA destruction and leakage into plasma (Korzeneva et al., 2015; Korzeneva et al., 2016). As such, the reduction of ribosome RNA may also be attributed the direct physical effects of radiation.

Our study has yielded a significant discovery, namely, that genes along the ribosome pathway were collectively downregulated in RNA-seq but upregulated in ATAC-seq. Gene expression is more accurately measured by RNA sequencing (RNA-seq), although ATAC-seq can explain the mechanism behind how gene expression is regulated or why it might be different between two cell types or conditions. Therefore, we believed that the expression of ribosomal protein-related genes was downregulated. To investigate the specific variations in ribosomal pathway between the two sequences, we examined the specific gene names and numbers in both datasets (Table 6; Supplementary Table S4) and found no overlap in genes. We postulated that the open peak regions in ATAC-seq were situated in the negative regulatory region near the ribosomal structural genes (Mansisidor and Risca, 2022), which impeded their upregulation. The aforementioned observations suggested that neutron-γ rays may not directly impact mRNAs but instead, regulated chromatin openness, which triggered the movement of regulatory genes and nucleosomes. This allowed other regulatory mechanisms to participate, leading to the collective downregulation of 63 ribosomal RNAs. Our study also revealed a surprising phenomenon that most of the ribosomal upregulated genes in ATAC-seq were pseudogenes, and the parental genes of some pseudogenes appeared in the set of genes downregulated in RNA-seq, such as RPS20P12 and RPS20P5 shared the parental gene RPS20 downregulated in RNA-seq (Figure 6C). As research on pseudogenes continues to expand, it is now widely accepted that these non-coding genes have the ability to regulate their parental genes. They can negatively modulate the transcriptional activity of their parents by transcribing into the antisense strand, generating endogenous siRNAs, and competitively binding micRNAs of the parental gene, etc (Poliseno et al., 2010; Korneev et al., 2013; Chan and Chang, 2014; Khan et al., 2015; Mahmudi et al., 2015; Grander and Johnsson, 2016; Kerwin et al., 2020; Fan and Ding, 2023). Based on these findings, we propose that neutron-γ radiation has a considerable stimulatory effect on ribosomal pseudogenes, eventually leading to the upregulation of these pseudogenes and subsequent downregulation of associated RNAs.

Genes coding for ribosomal proteins are relatively stably expressed in eukaryotic cells and even served as internal reference genes in qPCR experiments (Tao et al., 2020; Zhou et al., 2021), but the genes were also up- or downregulated in response to stress treatments (Moin et al., 2016). In the present sequencing, clusters of genes coding for ribosomal proteins were found to be downregulated in irradiated human lymphocytes, and it was these genes that responded to neutron radiation. Unlike photon radiation, which affects mitochondria (Cho et al., 2018), neutron radiation may specifically affect ribosomes and be manifested by differential expression of a large number of ribosomal genes. The number of downregulated ribosomal genes identified in this sequencing study is higher than those reported in previous experiments (Broustas et al., 2017b; Broustas et al., 2018; Mukherjee et al., 2019), which may be attributed to the selection of workers who have been exposed to radiation for a prolonged period. The genetic alterations in stimulated lymphocytes are likely to be more persistent and complex. This finding further supports the validity of previous experimental observations, highlighting the sensitivity and sustained response of ribosomal genes to neutron radiation. The qPCR experiments we performed confirmed that the mRNA expression of RPS18 and RPS27A in human lymphocytes was significantly downregulated after neutron-γ radiation. The expression of the RPS18 and RPS27A gene declined rapidly after radiation, but the trend of decline leveled off with increasing radiation dose(Figure 7), indicating that the gene was sensitive to neutron radiation but not to dose variation, at least within a certain dose range. It is understandable that the genes coding for ribosomal proteins was regulated after neutron radiation. Some experiments have confirmed that RPs was closely related to the changes of many radiation-related genes, such as P53, which mediates cell apoptosis or cycle arrest after radiation (Fumagalli et al., 2012; Li et al., 2020; Li et al., 2022; Luan et al., 2022). It can be seen that RPs is widely involved in the subsequent regulation of cells after radiation, and they are genes worthy of attention.

To further investigate the core genes involved, we utilized the STRING platform to generate PPI graphs for all differentially expressed genes and those in the ribosome pathway, respectively (Figures 8A1-8B2). Functional cluster analysis showed that the different genes were clearly clustered in ribosome biogenesis and translation. Notably, RPS18 was present in both intergraphs with high connectivity to other proteins, suggesting it might be a key protein influencing the entire pathway. Ribosomal proteins may play an unexpected role in the response to neutron-γ radiation.

Previous radiation experiments have demonstrated that radiation preferentially impacted translation-related genes over transcription-related genes (Lu et al., 2006; Hayman et al., 2012; Lu et al., 2016). In this experiment, we aimed to investigate whether the downregulation of ribosome mRNA leads to a corresponding decrease in protein levels. To address this question, we employed flow cytometry to examine the expression levels of RPS18 and RPS27A in human lymphocytes following neutron-γ gradient radiation. Surprisingly, our findings revealed that the expression of these ribosomal proteins did not align with changes in mRNA levels. This discrepancy suggests that radiation exerts a distinct effect on the translation process. These results are consistent with previous reports in the literature, in which changes in mRNA expression were not pronounced, translational regulation is nevertheless active (Tanenbaum et al., 2015). This phenomenon of mRNAs and proteins going in opposite directions has been interpreted in some literature to mean that the cell inhibits the transcription to conserve energy, with which to generate proteins in response to emergencies. Upon stimulation by radiation, ribosomal proteins are regulated and dynamically altered through multiple pathways such as SAPK/JNK 1, RAS, etc (Iordanov et al., 1998; Iordanov et al., 2002; Stumpf et al., 2013), RPS18 expression rises rapidly after radiation and appears to be sensitive to neutron-γ radiation, but does not increase with increasing dose, although it remains at a high level. Thus its expression results can suggest radiation exposure, but cannot yet be linked to the amount of dose. The expression of RPS27A, a component of the large subunit of the ribosome, was significantly affected by more factors in the gradient dose radiation experiment. Previously, it was reported that RPS27A has a reciprocal inhibition effect with P53 through RPMPS-1/S27 (Dressman et al., 2007; Fernandez-Pol, 2011), whereas after the cells were irradiated, P53 must be expressed in large quantities to block the cell cycle, which may contribute to the downregulation of RPS27A. In our experiments, the cell cycle was also examined after gradient-dose radiation, and it was found that the G2 phase of the lymphocytes, which were not proliferating actively, was gradually shortened with the increase of the irradiated dose, suggesting that pre-G2 phase arrest may have occurred (Figure 9), and that the cycle-associated proteins P21 and CDK2 were also changed, even though at relatively low irradiated doses. This gives us reason to believe that RPS27A may be involved in cell cycle regulation; after all, Ribo-seq has demonstrated that deficiencies in ribosomal proteins on the large subunit enhance the influence of P53 on the cell cycle (Luan et al., 2022). Additionally, other experiments have corroborated the extensive involvement of ribosomal proteins in cell cycle processes (Tanenbaum et al., 2015). Moreover, in our experiment, neutron-γ radiation induced concurrent abnormalities in proteins on both the large and small subunits of the ribosome, which may lead to an elevation in P53 levels and subsequent cell cycle arrest (Fumagalli et al., 2012; Li et al., 2020). Therefore, ribosomal proteins are closely related to cell cycle and cycle-related proteins. Overall, after neutron-γ radiation of cells, ribosomal DNA, RNA, and ribosomal proteins all responded to radiation and joined the intracellular adjustment process.

We also believed that by changing the type and dose of radiation, the changes in gene regulation might differ from our results. Previous experiments suggested that each different level of radiation exposure induced a unique hierarchy of transcriptional events, rather than an escalating biological response from a select group of genes (Brabcova et al., 2019; Dahl et al., 2023).

This experiment presented both advantages and limitations. The direct sourcing of cells from human subjects was advantageous for exploring the mechanism of human cellular radiation response and might shed light on radiation-induced diseases. Moreover, the rapid sequencing of live cells without culturing maximized the *in vivo* gene expression status of the cells. Furthermore, using the same sample for both ATAC-se and RNA-seq enabled a more direct and accurate comparison of the two results. Previous radiation biology experiments have confirmed that radiation tends to damage DNA with dense structures and nucleic acid aggregates (Banerjee et al., 2019; Akamatsu et al., 2021; Wang et al., 2021), Therefore, the results of ATAC sequencing provide a wealth of information for identifying regulatory mechanisms at the source. The combination of RNA sequencing and ATAC sequencing is a perfect approach to explore the interplay between DNA and RNA. However, the experiment was limited in that lymphocyte subtypes were not differentiated, which hinders the understanding of the mechanism and extent of response of different lymphocyte subtypes. Additionally, due to difficulties in obtaining human samples and financial support, quantitative analysis of cellular gene expression in workers receiving doses was not possible.

In summary, we present a novel resource for identifying open chromatin regions in human lymphocytes. Our findings demonstrate that integrating ATAC-seq with RNA-seq enhances the interpretation of computational results and provides insight into the tightly regulated gene expression networks. We anticipate that these datasets will prove useful for future genomic analyses of human lymphocytes.

## 5 Conclusion

Neutron-γ radiation has been shown to regulate the opening of specific gene regions and alter gene expression at the DNA and RNA levels, resulting in the downregulation of both chromatin opening and gene expression. The most significant response to neutron-γ radiation was the differential expression of ribosome-related genes. While open chromatin regions and differential RNA expression by neutron-γ radiation might be in the same pathway, they did not exhibit a simple one-to-one correspondence. Rather, there existed a complex regulatory mechanism between the two, in which ribosomal pseudogenes might suppress parental genes.

## Data Availability

The raw sequence data reported in this paper have been deposited in the Genome Sequence Archive (Genomics, Proteomics & Bioinformatics 2021) in National Genomics Data Center (Nucleic Acids Res 2022), China National Center for Bioinformation / Beijing Institute of Genomics, Chinese Academy of Sciences (GSA-Human: HRA006039 for RNA-seq and HRA006040 for ATAC-seq) that are publicly accessible at https://ngdc.cncb.ac.cn/gsa-human.

## Supplementary material

Supplementary Figure S1 The distribution of fragments obtained from each sample is almost identical in RNA-seq. **(A)** Correlation coefficient matrix of reads between samples for RNA-seq. **(B)** Violin diagram of FPKM distribution in each sample in RNA-seq sequencing. The Y-axis showed the FPKM value, and the violin width showed the relative amount of genes with a certain FPKM.

Supplementary Figure S2 The figure illustrated a top-down inclusion relationship of functions in a DAG (Directed Acyclic Graph). The boxes represented the top 10 GO enrichments, with darker colors indicating higher enrichment. The branches represented the inclusion relationship, with the functional range narrowing from top to bottom. The figure specifically displayed the enrichment terms in the cellular component.

Supplementary Figure S3 The figure illustrated a top-down inclusion relationship of functions in a DAG (Directed Acyclic Graph). The boxes represented the top 10 GO enrichments, with darker colors indicating higher enrichment. The branches represented the inclusion relationship, with the functional range narrowing from top to bottom. The figure specifically displayed the down-regulated enrichment terms in the cellular component.

Supplementary Figure S4 The fold enrichment and location of reads obtained from each sample was almost identical in ATAC-seq. **(A)** Pearson corralations of foldenrich in peaks of samples. **(B)** Peak width distribution.

Supplementary Figure S5 The distribution of reads obtained from each sample was almost identical in ATAC-seq. **(A)** Peak width distribution. **(B)** reads distribution to TSS in all samples. **(C)** Peaks annotation distribution in different gene functional regions.

## References

[B1] Akamatsu K. Shikazono N. Saito T. (2021). Fluorescence anisotropy study of radiation-induced DNA damage clustering based on FRET. Anal. Bioanal. Chem. 413 (4), 1185–1192. 10.1007/s00216-020-03082-w 33245399

[B2] Banerjee S. Selim M. Saha A. Mukherjea K. K. (2019). Radiation induced DNA damage and its protection by a gadolinium(III) complex: spectroscopic, molecular docking and gel electrophoretic studies. Int. J. Biol. Macromol. 127, 520–528. 10.1016/j.ijbiomac.2019.01.031 30633933

[B3] Bouten R. M. Dalgard C. L. Soltis A. R. Slaven J. E. Day R. M. (2021). Transcriptomic profiling and pathway analysis of cultured human lung microvascular endothelial cells following ionizing radiation exposure. Sci. Rep-Uk 11 (1), 24214. 10.1038/s41598-021-03636-7 PMC8688546 34930946

[B4] Brabcova K. P. Jamborova Z. Michaelidesova A. Davidkova M. Kodaira S. Sefl M. (2019). Radiation-induced plasmid DNA damage: effect of concentration and length. Radiat. Prot. Dosim. 186 (2-3), 168–171. 10.1093/rpd/ncz196 31803909

[B5] Broerse J. J. Barendsen G. W. van Kersen G. R. (1968). Survival of cultured human cells after irradiation with fast neutrons of different energies in hypoxic and oxygenated conditions. Int. J. Radiat. Biol. Relat. Stud. Phys. Chem. Med. 13 (6), 559–572. 10.1080/09553006814550621 5301983

[B6] Broustas C. G. Harken A. D. Garty G. Amundson S. A. (2018). Identification of differentially expressed genes and pathways in mice exposed to mixed field neutron/photon radiation. Bmc Genomics 19 (1), 504. 10.1186/s12864-018-4884-6 29954325 PMC6027792

[B7] Broustas C. G. Xu Y. Harken A. D. Chowdhury M. Garty G. Amundson S. A. (2017a). Impact of neutron exposure on global gene expression in a human peripheral blood model. Radiat. Res. 187 (4), 443. 10.1667/RR0005.1 PMC5525057 28140791

[B8] Broustas C. G. Xu Y. Harken A. D. Garty G. Amundson S. A. (2017b). Comparison of gene expression response to neutron and x-ray irradiation using mouse blood. Bmc Genomics 18 (1), 2. 10.1186/s12864-016-3436-1 28049433 PMC5210311

[B9] Buenrostro J. D. Giresi P. G. Zaba L. C. Chang H. Y. Greenleaf W. J. (2013). Transposition of native chromatin for fast and sensitive epigenomic profiling of open chromatin, DNA-binding proteins and nucleosome position. Nat. Methods 10 (12), 1213–1218. 10.1038/nmeth.2688 24097267 PMC3959825

[B10] Chan W. L. Chang J. G. (2014). Pseudogene-derived endogenous siRNAs and their function. Methods Mol. Biol. 1167, 227–239. 10.1007/978-1-4939-0835-6_15 24823781

[B11] Cho S. J. Kang H. Hong E. H. Kim J. Y. Nam S. Y. (2018). Transcriptome analysis of low-dose ionizing radiation-impacted genes in CD4(+) T-cells undergoing activation and regulation of their expression of select cytokines. J. Immunotoxicol. 15 (1), 137–146. 10.1080/1547691X.2018.1521484 30686136

[B12] Dahl H. Ballangby J. Tengs T. Wojewodzic M. W. Eide D. M. Brede D. A. (2023). Dose rate dependent reduction in chromatin accessibility at transcriptional start sites long time after exposure to gamma radiation. Epigenetics-Us. 18 (1), 2193936. 10.1080/15592294.2023.2193936 PMC10054331 36972203

[B13] Dressman H. K. Muramoto G. G. Chao N. J. Meadows S. Marshall D. Ginsburg G. S. (2007). Gene expression signatures that predict radiation exposure in mice and humans. Plos Med. 4 (4), e106. 10.1371/journal.pmed.0040106 17407386 PMC1845155

[B14] Durante M. (2014). New challenges in high-energy particle radiobiology. Br. J. Radiology 87 (1035), 20130626. 10.1259/bjr.20130626 PMC4064605 24198199

[B15] Fan C. Ding M. (2023). Inferring pseudogene–MiRNA associations based on an ensemble learning framework with similarity kernel fusion. Sci. Rep-Uk 13 (1), 8833. 10.1038/s41598-023-36054-y PMC10232424 37258695

[B16] Fernandez-Pol J. A. (2011). Conservation of multifunctional ribosomal protein metallopanstimulin-1 (RPS27) through complex evolution demonstrates its key role in growth regulation in Archaea, eukaryotic cells, DNA repair, translation and viral replication. Cancer Genom Proteom 8 (3), 105–126. 21518817

[B17] Fumagalli S. Ivanenkov V. V. Teng T. Thomas G. (2012). Suprainduction of p53 by disruption of 40S and 60S ribosome biogenesis leads to the activation of a novel G2/M checkpoint. Gene Dev. 26 (10), 1028–1040. 10.1101/gad.189951.112 22588717 PMC3360559

[B18] Grander D. Johnsson P. (2016). Pseudogene-expressed RNAs: emerging roles in gene regulation and disease. Curr. Top. Microbiol. 394, 111–126. 10.1007/82_2015_442 25982975

[B19] Grandt C. L. Brackmann L. K. Foraita R. Schwarz H. Hummel-Bartenschlager W. Hankeln T. (2023). Gene expression variability in long-term survivors of childhood cancer and cancer-free controls in response to ionizing irradiation. Mol. Med. 29 (1), 41. 10.1186/s10020-023-00629-2 36997855 PMC10061869

[B20] Hayman T. J. Williams E. S. Jamal M. Shankavaram U. T. Camphausen K. Tofilon P. J. (2012). Translation initiation factor eIF4E is a target for tumor cell radiosensitization. Cancer Res. 72 (9), 2362–2372. 10.1158/0008-5472.CAN-12-0329 22397984 PMC6327305

[B21] Holden S. Perez R. Hall R. Fallgren C. M. Ponnaiya B. Garty G. (2021). Effects of acute and chronic exposure to a mixed field of neutrons and photons and single or fractionated simulated galactic cosmic ray exposure on behavioral and cognitive performance in mice. Radiat. Res. 196 (1), 31–39. 10.1667/RADE-20-00228.1 33857301 PMC8297553

[B22] Information needed to make radiation protection recommendations for space missions beyond low-earth orbit. Bethesda, MD: National Council on Radiation Protection and Measurements; 2006.

[B23] Iordanov M. S. Choi R. J. Ryabinina O. P. Dinh T. H. Bright R. K. Magun B. E. (2002). The UV (Ribotoxic) stress response of human keratinocytes involves the unexpected uncoupling of the Ras-extracellular signal-regulated kinase signaling cascade from the activated epidermal growth factor receptor. Mol. Cell Biol. 22 (15), 5380–5394. 10.1128/MCB.22.15.5380-5394.2002 12101233 PMC133934

[B24] Iordanov M. S. Pribnow D. Magun J. L. Dinh T. H. Pearson J. A. Magun B. E. (1998). Ultraviolet radiation triggers the ribotoxic stress response in mammalian cells. J. Biol. Chem. 273 (25), 15794–15803. 10.1074/jbc.273.25.15794 9624179

[B57] Kanehisa M. Araki M. Goto S. Hattori M. Hirakawa M. Itoh M. (2007). KEGG for linking genomes to life and the environment. Nucleic Acids Res. 36, D480–D484. 10.1093/nar/gkm882 18077471 PMC2238879

[B25] Kerwin J. Khan I. Iorns E. Tsui R. Denis A. Perfito N. (2020). Replication Study: a coding-independent function of gene and pseudogene mRNAs regulates tumour biology. Elife 9, e51019. 10.7554/eLife.51019 32314732 PMC7185998

[B26] Khan I. Kerwin J. Owen K. Griner E. Reproducibility Project: Cancer Biology (2015). Correction: registered report: a coding-independent function of gene and pseudogene mRNAs regulates tumour biology. Elife 4, e11802. 10.7554/eLife.11802 26599840 PMC4656975

[B27] Korneev S. A. Kemenes I. Bettini N. L. Kemenes G. Staras K. Benjamin P. R. (2013). Axonal trafficking of an antisense RNA transcribed from a pseudogene is regulated by classical conditioning. Sci. Rep-Uk 3, 1027. 10.1038/srep01027 PMC3537157 23293742

[B28] Korzeneva I. B. Kostuyk S. V. Ershova E. S. Skorodumova E. N. Zhuravleva V. F. Pankratova G. V. (2016). Human circulating ribosomal DNA content significantly increases while circulating satellite III (1q12) content decreases under chronic occupational exposure to low-dose gamma-neutron and tritium beta-radiation. Mutat. Res-Fund Mol. M. 791-792, 49–60. 10.1016/j.mrfmmm.2016.09.001 27648955

[B29] Korzeneva I. B. Kostuyk S. V. Ershova L. S. Osipov A. N. Zhuravleva V. F. Pankratova G. V. (2015). Human circulating plasma DNA significantly decreases while lymphocyte DNA damage increases under chronic occupational exposure to low-dose gamma-neutron and tritium beta-radiation. Mutat. Res-Fund Mol. M. 779, 1–15. 10.1016/j.mrfmmm.2015.05.004 26113293

[B30] Li H. Zhang H. Huang G. Bing Z. Xu D. Liu J. (2022). Loss of RPS27a expression regulates the cell cycle, apoptosis, and proliferation via the RPL11-MDM2-p53 pathway in lung adenocarcinoma cells. J. Exp. Clin. Canc Res. 41 (1), 33. 10.1186/s13046-021-02230-z PMC8785590 35073964

[B31] Li H. Zhang H. Huang G. Dou Z. Xie Y. Si J. (2020). Heavy ion radiation-induced DNA damage mediates apoptosis via the Rpl27a-Rpl5-MDM2-p53/E2F1 signaling pathway in mouse spermatogonia. Ecotox Environ. Safe 201, 110831. 10.1016/j.ecoenv.2020.110831 32535367

[B32] Little M. P. (2018). Evidence for dose and dose rate effects in human and animal radiation studies. Ann. Icrp 47 (3-4), 97–112. 10.1177/0146645318756235 29652168

[B33] Lu C. Makala L. Wu D. Cai Y. (2016). Targeting translation: eIF4E as an emerging anticancer drug target. Expert Rev. Mol. Med. 18, e2. 10.1017/erm.2015.20 26775675

[B34] Lu X. de la Pena L. Barker C. Camphausen K. Tofilon P. J. (2006). Radiation-induced changes in gene expression involve recruitment of existing messenger RNAs to and away from polysomes. Cancer Res. 66 (2), 1052–1061. 10.1158/0008-5472.CAN-05-3459 16424041

[B35] Luan Y. Tang N. Yang J. Liu S. Cheng C. Wang Y. (2022). Deficiency of ribosomal proteins reshapes the transcriptional and translational landscape in human cells. Nucleic Acids Res. 50 (12), 6601–6617. 10.1093/nar/gkac053 35137207 PMC9262593

[B36] Mahmudi O. Sennblad B. Arvestad L. Nowick K. Lagergren J. (2015). Gene-pseudogene evolution: a probabilistic approach. Bmc Genomics 16 (10), S12. 10.1186/1471-2164-16-S10-S12 PMC4602177 26449131

[B37] Mansisidor A. R. Risca V. I. (2022). Chromatin accessibility: methods, mechanisms, and biological insights. Nucleus-Phila. 13 (1), 238–278. 10.1080/19491034.2022.2143106 PMC9683059 36404679

[B38] Moin M. Bakshi A. Saha A. Dutta M. Madhav S. M. Kirti P. B. (2016). Rice ribosomal protein large subunit genes and their spatio-temporal and stress regulation. Front. Plant Sci. 7, 1284. 10.3389/fpls.2016.01284 27605933 PMC4995216

[B39] Moriyama H. Daino K. Imaoka T. Nishimura M. Nishimura Y. Takabatake M. (2019). Neutron-induced rat mammary carcinomas are mainly of luminal subtype and have multiple copy number aberrations. Anticancer Res. 39 (3), 1135–1142. 10.21873/anticanres.13222 30842142

[B40] Mukherjee S. Grilj V. Broustas C. G. Ghandhi S. A. Harken A. D. Garty G. (2019). Human transcriptomic response to mixed neutron-photon exposures relevant to an improvised nuclear device. Radiat. Res. 192 (2), 189. 10.1667/RR15281.1 31237816 PMC7450517

[B41] Neary G. J. Evans H. J. Tonkinson S. M. Williamson F. S. (1959). The relative biological efficiency of single doses of fast neutrons and gamma-rays on Vicia faba roots and the effect of oxygen. Part III. Mitotic delay. Int. J. Radiat. Biol. Relat. Stud. Phys. Chem. Med. 1, 230–240. 10.1080/09553005914550321 14426278

[B42] Poliseno L. Salmena L. Zhang J. Carver B. Haveman W. J. Pandolfi P. P. (2010). A coding-independent function of gene and pseudogene mRNAs regulates tumour biology. Nature 465 (7301), 1033–1038. 10.1038/nature09144 20577206 PMC3206313

[B43] Schmidt T. Dabrowska A. Waldron J. A. Hodge K. Koulouras G. Gabrielsen M. (2023). eIF4A1-dependent mRNAs employ purine-rich 5'UTR sequences to activate localised eIF4A1-unwinding through eIF4A1-multimerisation to facilitate translation. Nucleic Acids Res. 51 (4), 1859–1879. 10.1093/nar/gkad030 36727461 PMC9976904

[B44] Sekihara K. Saitoh K. Yang H. Kawashima H. Kazuno S. Kikkawa M. (2018). Low-dose ionizing radiation exposure represses the cell cycle and protein synthesis pathways in *in vitro* human primary keratinocytes and U937 cell lines. Plos One 13 (6), e0199117. 10.1371/journal.pone.0199117 29912936 PMC6005503

[B45] Shimabukuro K. Fukazawa T. Kanai A. Kawai H. Mekata K. Hirohashi N. (2022). Low-dose-rate irradiation suppresses the expression of cell cycle-related genes, resulting in modification of sensitivity to anti-cancer drugs. Cells-Basel 11 (3), 501. 10.3390/cells11030501 PMC8833988 35159310

[B46] Stichelbaut F. Closset M. Jongen Y. (2014). Secondary neutron doses in a compact proton therapy system. Radiat. Prot. Dosim. 161 (1-4), 368–372. 10.1093/rpd/ncu028 24591728

[B47] Stumpf C. R. Moreno M. V. Olshen A. B. Taylor B. S. Ruggero D. (2013). The translational landscape of the mammalian cell cycle. Mol. Cell 52 (4), 574–582. 10.1016/j.molcel.2013.09.018 24120665 PMC3959127

[B48] Tanenbaum M. E. Stern-Ginossar N. Weissman J. S. Vale R. D. (2015). Regulation of mRNA translation during mitosis. Elife 4, e07957. 10.7554/eLife.07957 26305499 PMC4548207

[B49] Tao J. Hao Y. Li X. Yin H. Nie X. Zhang J. (2020). Systematic identification of housekeeping genes possibly used as references in *Caenorhabditis elegans* by large-scale data integration. Cells-Basel 9 (3), 786. 10.3390/cells9030786 PMC7140892 32213971

[B50] Tsompana M. Buck M. J. (2014). Chromatin accessibility: a window into the genome. Epigenet Chromatin 7 (1), 33. 10.1186/1756-8935-7-33 PMC4253006 25473421

[B51] Tsoulou E. Kalfas C. A. Conformational E. G. (2005). Conformational properties of DNA after exposure to gamma rays and neutrons. Radiat. Res. 163 (1), 90–97. 10.1667/rr3274 15606312

[B52] Wang Q. Lee Y. Pujol-Canadell M. Perrier J. R. Smilenov L. Harken A. (2021). Cytogenetic damage of human lymphocytes in humanized mice exposed to neutrons and X rays 24 h after exposure. Cytogenet Genome Res. 161 (6-7), 352–361. 10.1159/000516529 34488220 PMC8455411

[B53] Wang Y. Bannister L. A. Sebastian S. Le Y. Ismail Y. Didychuk C. (2019). Low-dose radiobiology program at Canadian nuclear laboratories: past, present, and future. Int. J. Radiat. Biol. 95 (10), 1361–1371. 10.1080/09553002.2018.1562252 30582711

[B54] Xiao F. H. Yu Q. Deng Z. L. Yang K. Ye Y. Ge M. X. (2022). ETS1 acts as a regulator of human healthy aging via decreasing ribosomal activity. Sci. Adv. 8 (17), eabf2017. 10.1126/sciadv.abf2017 35476452 PMC9045719

[B56] Young M. D. Wakefield M. J. Smyth G. K. Oshlack A. (2010). Gene ontology analysis for RNA-seq: accounting for selection bias. Genome Biol. 11 (2), R14. 10.1186/gb-2010-11-2-r14 20132535 PMC2872874

[B55] Zhou L. Meng J. Y. Ruan H. Y. Yang C. L. Zhang C. Y. (2021). Expression stability of candidate RT-qPCR housekeeping genes inSpodoptera frugiperda (Lepidoptera: noctuidae). Arch. Insect Biochem. 108 (1), e21831. 10.1002/arch.21831 34240760

