# Supplementary figures and images for: Integration of RNA-seq and ATAC-seq analyzes the effect of low dose neutron-γ radiation on gene expression of lymphocytes from oilfield logging workers
Source: Front Chem. 2023 Nov 30;11:1269911. doi: 10.3389/fchem.2023.1269911 (PMC10720751; doi:10.3389/fchem.2023.1269911)

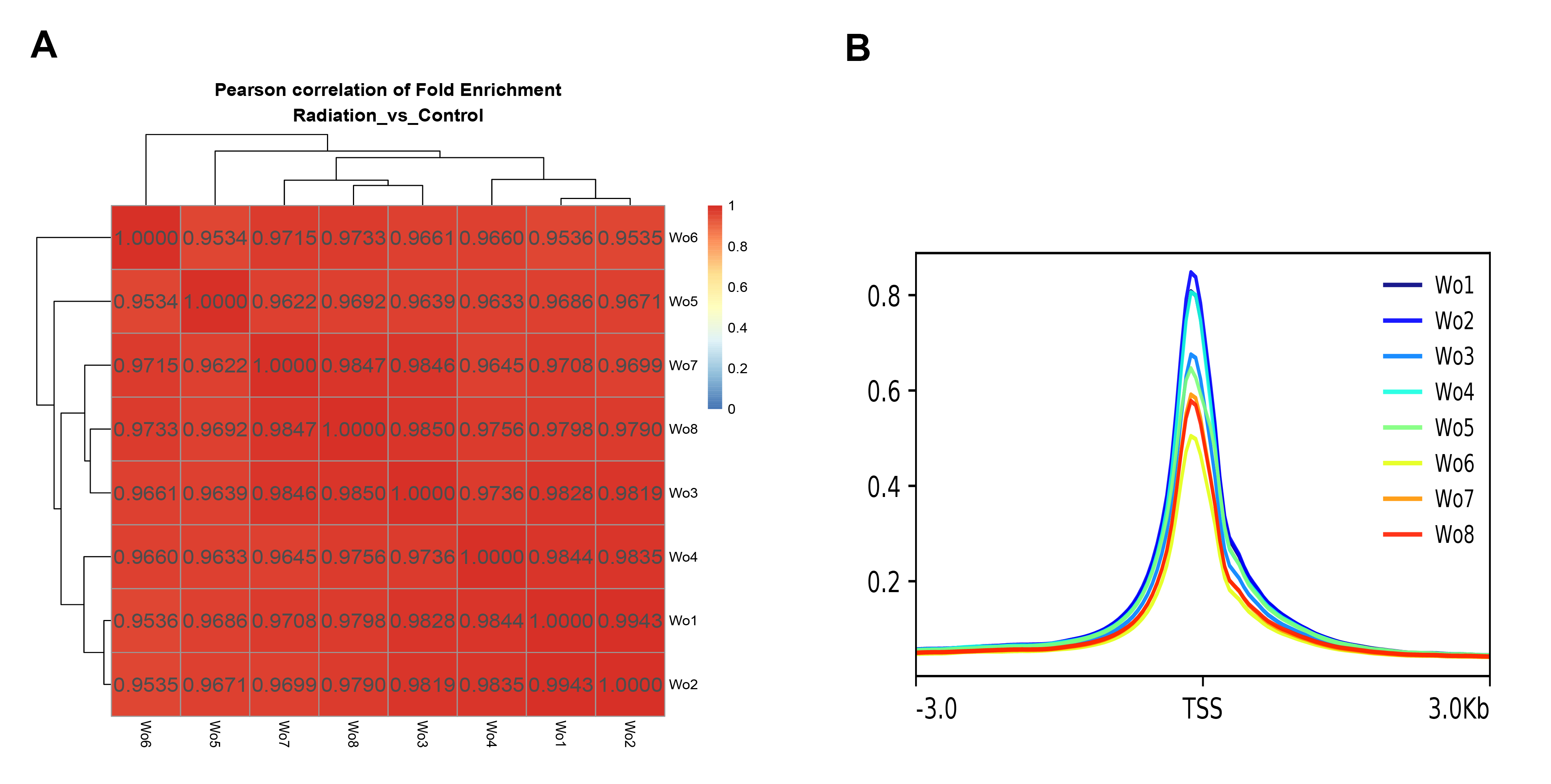

Supplement: Supplementary file 1 [file Image4.tif]

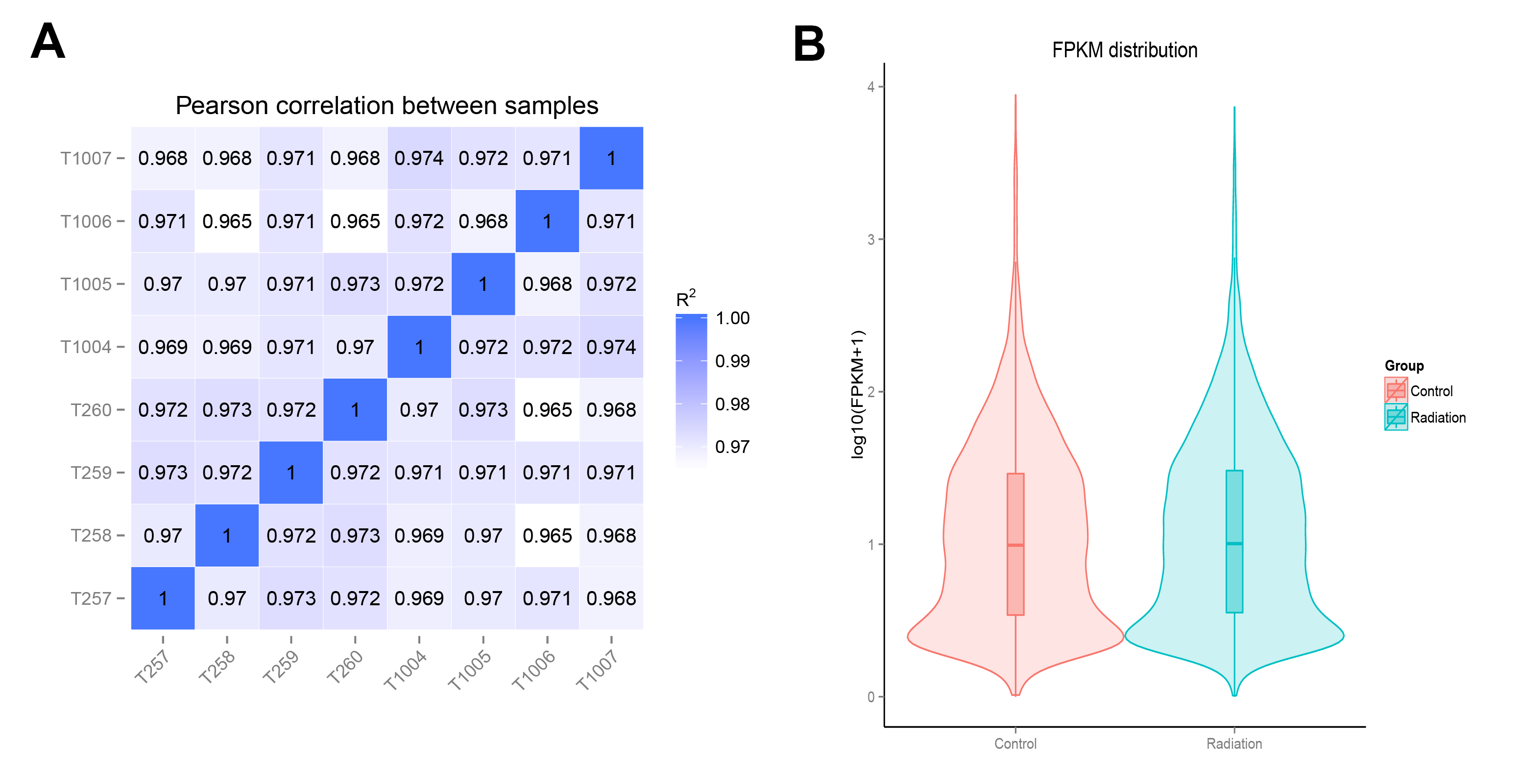

Supplement: Supplementary file 2 [file Image1.tif]

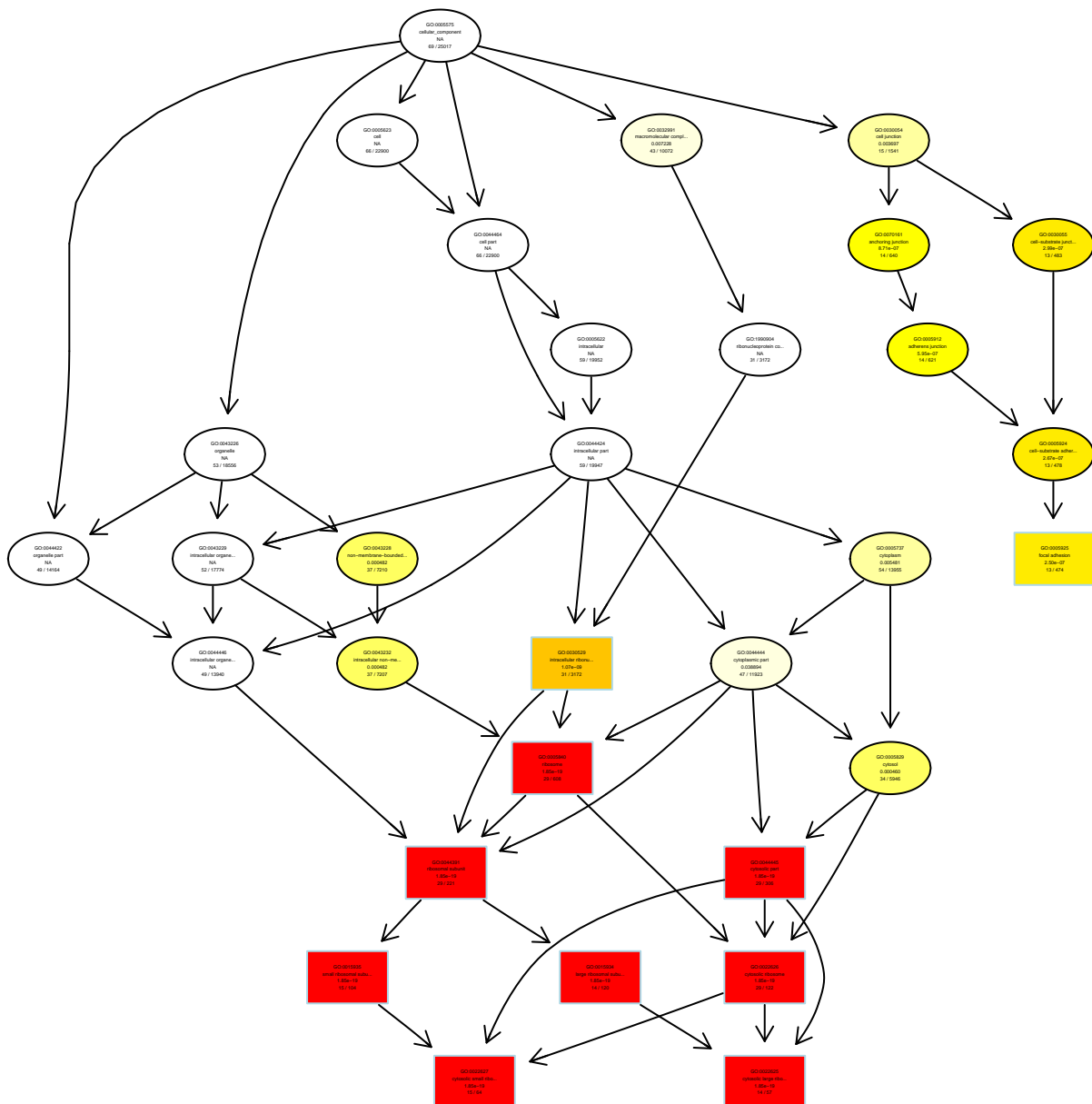

Supplement: Supplementary file 3 [file Image2.pdf]

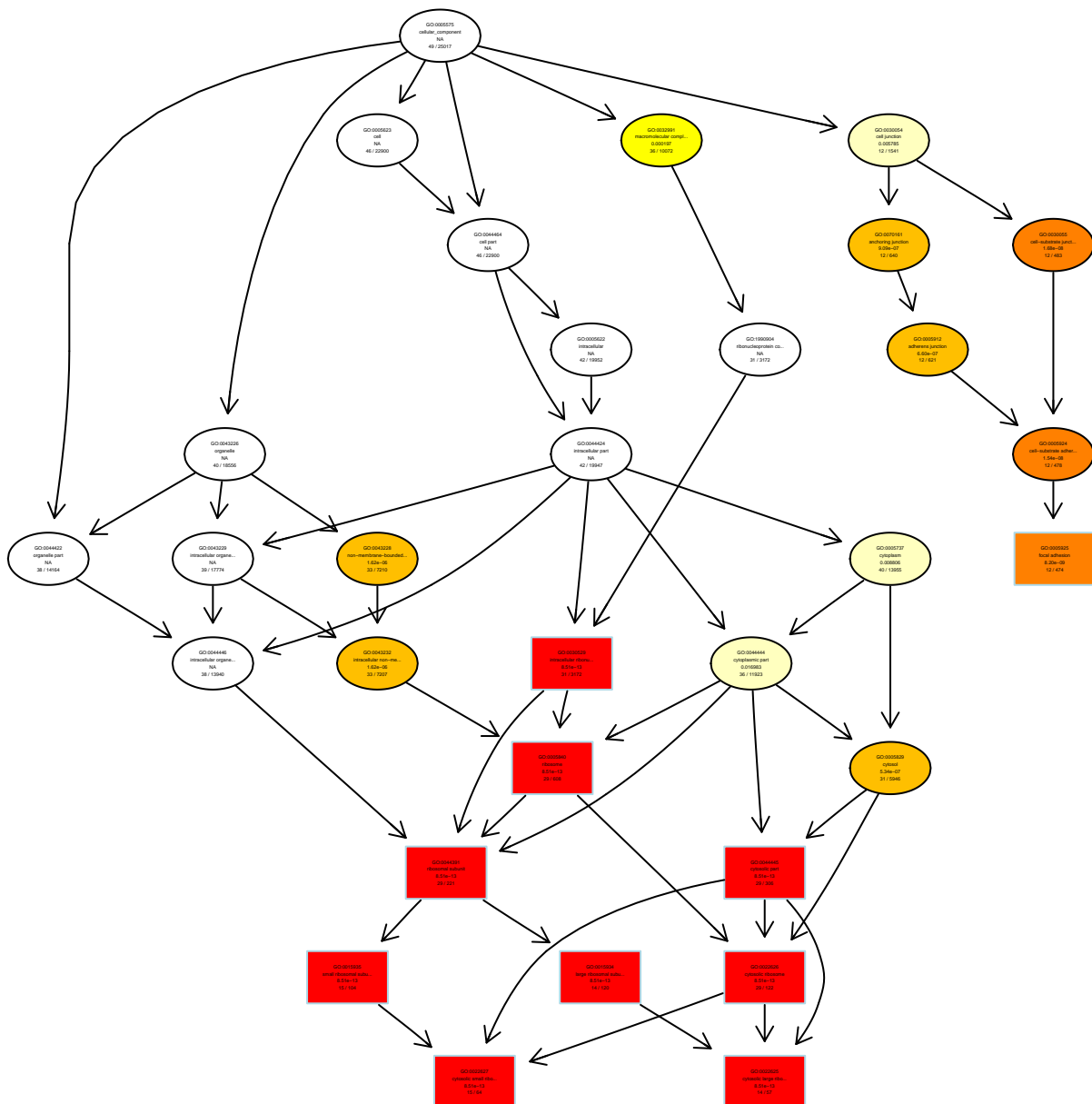

Supplement: Supplementary file 4 [file Image3.pdf]

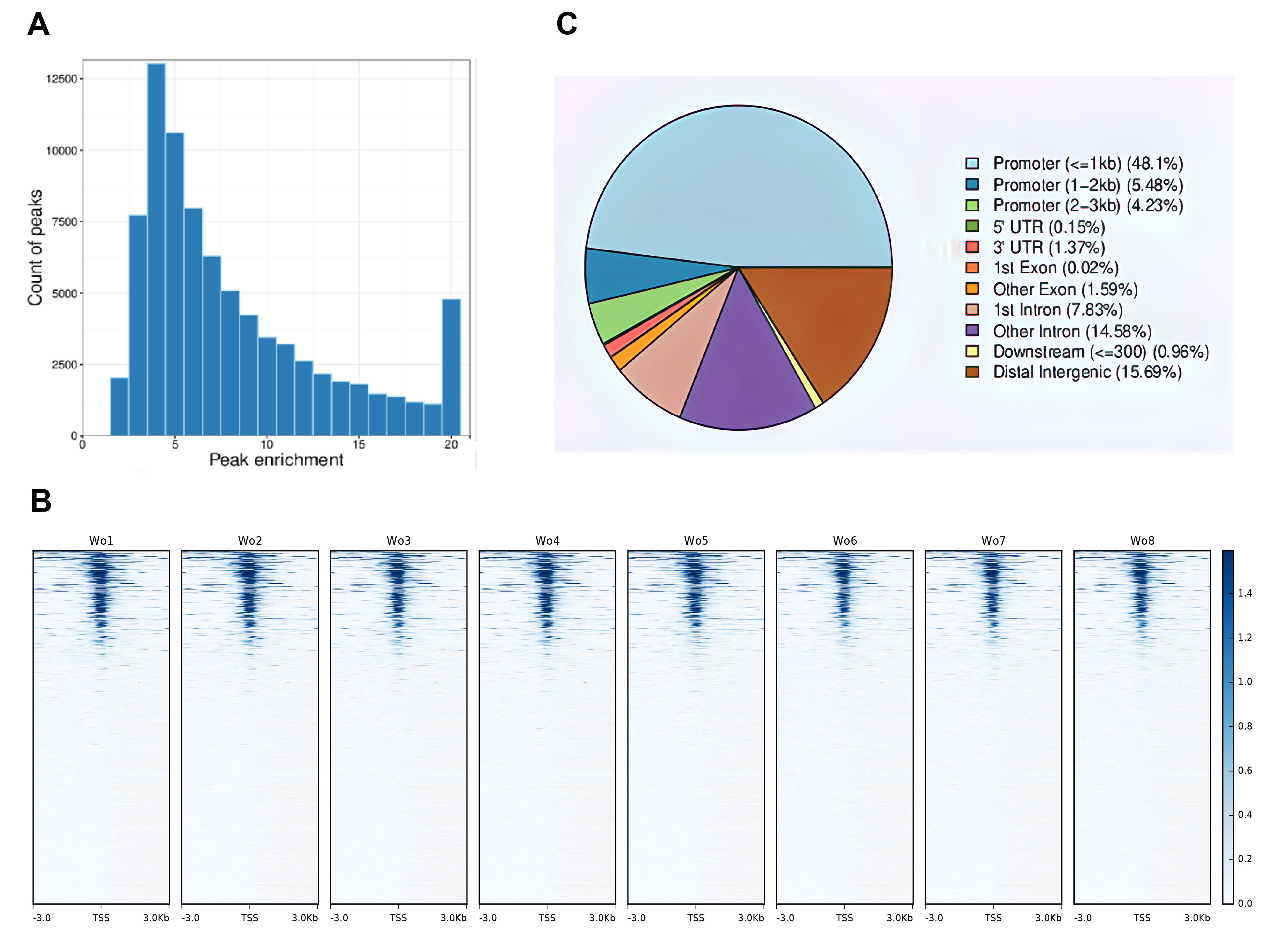

Supplement: Supplementary file 5 [file Image5.tif]
